# Base Metal Photocatalysts: A Bright Future in Photoredox Catalysis?

**DOI:** 10.1021/acs.organomet.5c00444

**Published:** 2026-03-06

**Authors:** Subrata Pal, Martin Pauze, Negin Shafiei, Spencer P. Pitre

**Affiliations:** Department of Chemistry, 7618 Oklahoma State University, Stillwater, Oklahoma 74078, United States

## Abstract

Photoredox catalysis is widely considered as a more sustainable alternative for performing chemical synthesis, using low-energy visible-light photons as traceless reagents to achieve the desired reactivity under mild conditions. While this has led to significant improvements in sustainability for many transformations, the environmental impact of the photocatalysts themselves is often overlooked, with many methods relying on precious metals with uncertain long-term availability. One solution that remains underexplored is the development of photocatalysts using earth-abundant base metals. This tutorial review discusses current strategies for designing effective base metal photocatalysts and highlights recent notable examples of their implementation in photoredox-catalyzed transformations.

## Graphical abstract

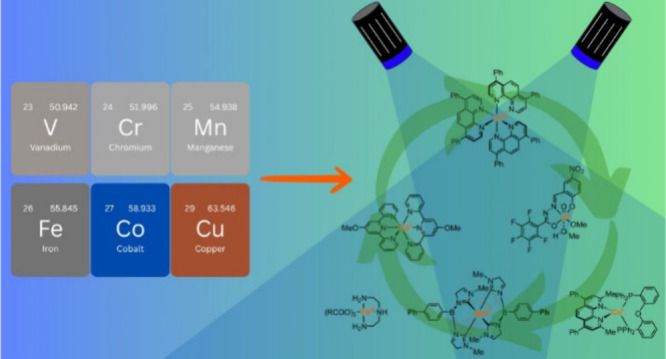

## 1 Introduction

### 1.1 Photoredox Catalysis and Sustainability

While the use of visible-light photochemistry in organic synthesis has seen a rejuvenation in interest in the last two decades, organic photochemistry can be dated back over a century to the laboratory of Giacomo Ciamician. In his famous article “The photochemistry of the future”, Ciamician challenged scientists to imagine a chemical industry that could synthesize chemicals in the same manner as plants: by using light, particularly sunlight, as a safe, abundant, inexpensive, and renewable energy source.^1^ An important step toward Ciamician’s goal has been the continued growth of the field of photoredox catalysis, which leverages visible-light absorbing photosensitizers to generate high-energy excited states capable of generating reactive radicals from low-energy reactants through single-electron transfer (SET) or energy transfer processes.^2–4^ This mild and efficient strategy for radical generation has led to a tremendous number of discoveries and novel organic transformations,^5^ addressing many longstanding challenges in, but not limited to, medicinal chemistry,^6–8^ natural product synthesis,^9–11^ cross-coupling reactions,^12,13^ and materials chemistry,^14,15^ which have been summarized in many excellent prior reviews.

Along with the impact photoredox catalysis has had on the organic chemistry community, it is widely considered as a more sustainable, or “greener”, alternative for chemical synthesis,^16,17^ fulfilling several of the Green Chemistry Principles.^18^ For example, the primary energy source for these methodologies is visible light. Photons can be considered as “traceless” reagents,^19^ capable of providing enough energy to achieve the desired reactivity under mild conditions and without producing any waste. This is often in contrast to the high temperatures or harsh conditions necessitated by reactions that require thermal activation. The photosensitizer is also used in catalytic amounts, satisfying another green chemistry principle. Upon visible-light-absorption, this creates a catalytic quantity of a high-energy excited state in a controlled manner that can be used for SET or energy transfer processes. This negates the requirement for using high-energy ground state reagents, providing less hazardous and safer synthetic routes. In addition, this also has the added benefit of the increased functional group tolerance that is observed in photoredox reactions.

While many of these features of photoredox catalysis indeed mark important steps toward a sustainable, light-mediated chemical industry, it is also important to understand the sustainability of these reactions cannot be solely ascribed to the use of light or catalytic photosensitizers, and a critical assessment of all aspects of the reaction is needed. For example, many photoredox reactions still require the use of stoichiometric sacrificial reagents, toxic solvents, or preactivated starting materials. Furthermore, the identity and preparation of the photosensitizers themselves are often ignored when evaluating the greenness of these protocols. In a recent review from Quintavalla, Carboni, and Lombardo, the authors critically evaluated the environmental impact of a series of photoredox-catalyzed reactions using more quantitative metrics such as process mass intensity (PMI).^20^ The authors cautioned that while employing catalysis is often used as a primary criterion for recognizing a process as sustainable, it often neglects the waste and cost associated with the preparation of the catalyst itself. In this regard, many of the common photocatalysts employed today were found to be lacking in terms of overall PMI, mostly due to the extensive use of solvents employed during both preparation and purification. Furthermore, the synthetic preparation of the most widely used transition-metal-based photosensitizers were found to be energy intensive and use precious metals such as ruthenium and iridium.^20^ While the use of a ruthenium or iridium photocatalyst increases the overall cost of these processes, the long-term availability of these metals must also be considered, with current estimates indicating that known reserves of both ruthenium and iridium will be depleted within the next 50 years.^21,22^ Two potential solutions to decrease our reliance on these precious metals include the development of organic photocatalysts, which have been the subject of prior reviews,^23–25^ or through the development of photocatalysts using more earth-abundant base metals.

### 1.2 Base Metals

Despite the upward trend in base metal-catalyzed reactions in recent years, the term “base metal” remains ambiguously defined in the literature, frequently resulting in uncertainty regarding which metals fall under this category. In its simplest definition, base metals are those that react with aqueous hydrochloric acid, such as iron, nickel, lead, cobalt, and zinc.^26^ In its broadest definition, base metals are simply considered as those which are abundant and inexpensive. A more comprehensive and clear definition of base metals was recently proposed by Drover and co-workers, defining base metals as those who possess natural abundancies in the Earth’s crust greater than 10 mg/kg, are sourced using more environmentally friendly procedures, are often readily oxidized, and can participate in both one- or two-electron chemistry.^27^ Given the relative abundance of base metals compared to ruthenium and iridium (see Figure 1),^28^ classified within “platinum group metals” by the U.S. Geological Survey, as well as the economic and sustainable advantages of base metals, why do ruthenium and iridium photocatalysts continue to dominate in the photoredox space? Their sustained use as preferred photocatalysts in organic reaction development stems from several key factors, including stability, predictable and tunable reactivity, and being able to pick from a broad selection of already well-characterized, commercially available complexes.^29,30^ Additionally, ruthenium and iridium complexes also benefit from efficient intersystem crossing and extended excited triplet state lifetimes, due to the heavy atom effect,^31^ increasing the probability for excited-state electron- or energy-transfer processes and thereby increasing reaction efficiency. To gain further traction and shift the balance away from the use of precious metals in photoredox-catalyzed processes, base metal photocatalysts must strive to make inroads in these key aforementioned areas.

**Figure 1:**
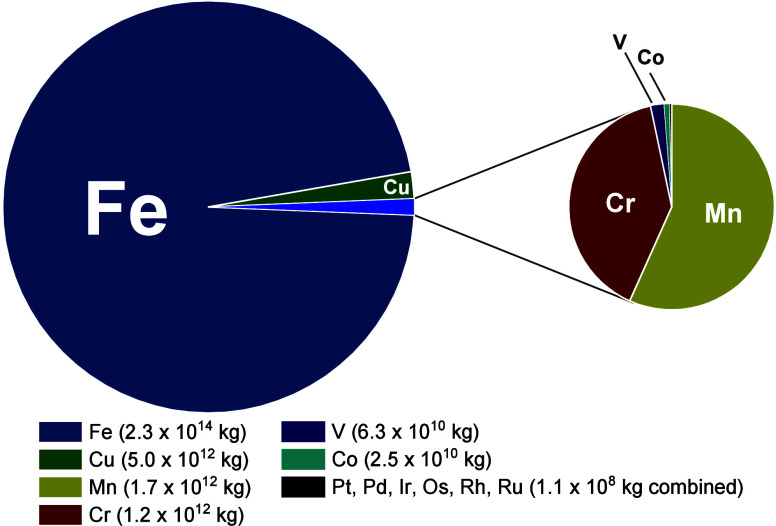
Relative amounts of estimated known world reserves for the base metals covered in this tutorial review compared to Pt group metals (Pt, Pd, Ir, Os, Rh, Ru) based on data from the 2025 Mineral Commodity Summaries from the U.S. Geological Survey.^28^

### 1.3 Base Metal Photocatalysis: Challenges and Current Strategies

Given the advantages of base metals in terms of cost, natural abundance, and long-term availability compared to platinum group metals, what factors continue to limit their widespread use in photoredox catalysis? One key drawback of base metal photosensitizers, specifically 3d metal complexes, is their short excited-state lifetimes. While the excited-state lifetimes for ruthenium and iridium polypyridyl complexes are generally on the microsecond scale, excited-state lifetimes for 3d base metal complexes are often within the femto- to picosecond range. Considering the efficiency of bimolecular excited-state quenching in photoinduced energy- and electron-transfer reactions is proportional to the magnitude of the excited-state lifetime of the photocatalyst, the impact on the overall kinetics of the reaction quickly becomes evident. The short excited-state lifetimes of 3d base metal photosensitizers are generally the result of two independent factors. The most predominant challenge is the decrease in ligand field strength when going from 4d or 5d metal to a 3d metal species, which is a consequence of the more contracted 3d orbitals compared to their 4d or 5d counterparts.^32,33^ As a result, base metal complexes with partially filled 3d orbitals typically possess multiple metal-centered (MC) excited states which are close in energy to one another and to the ground state (Scheme 1A).^34^ In this situation, excited states typically undergo rapid nonradiative decay, cascading from one MC state to another before returning to the ground state. Additionally, 3d base metal complexes generally possess a high degree of ligand lability,^35^ a feature which also contributes to their shorter excited-state lifetimes.

**Scheme 1:**
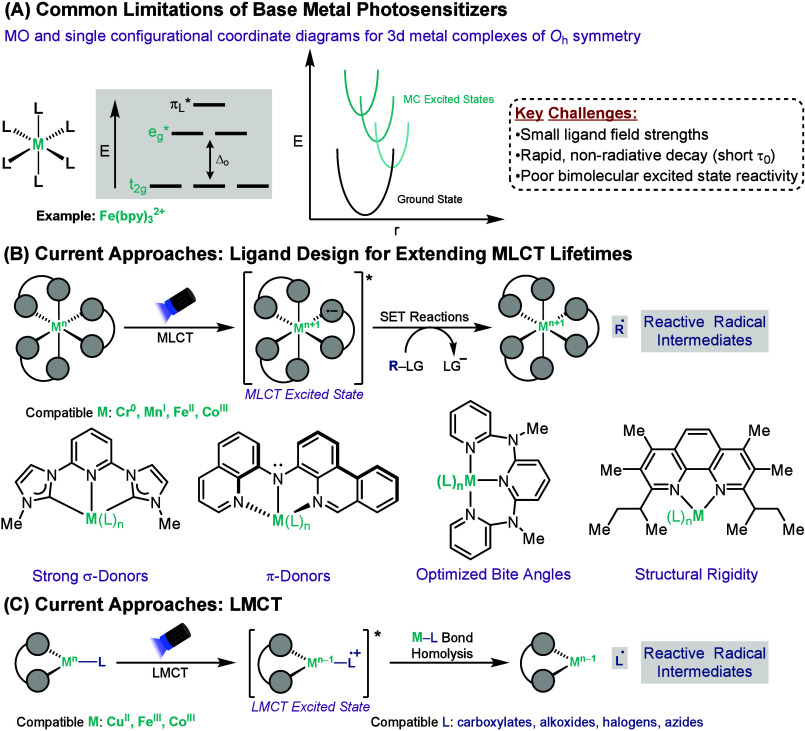
Limitations of Base Metal Photosensitizers and Current Approaches to Solutions

What strategies currently exist to overcome these challenges for base metal photocatalysts? These approaches can be divided into two categories: improving outer-sphere reactivity through ligand design or leveraging inner-sphere excited-state processes. The first approach involves strategies for improving the lifetime of metal-to-ligand charge transfer (MLCT), which arise from metal complexes with a metal-centered HOMO and a ligand-centered LUMO (Scheme 1B). Upon excitation, an electron is promoted from one of the d orbitals of the metal, often Cu^I^, Fe^II^, or Cr^0^, to a low-lying π* orbital of the ligand. In recent years, significant progress has been made in ligand design to help increase the lifetimes of MLCT excited states for base metal complexes.^33^ For example, the ligand field strength can be increased by employing strong σ donor ligands, such as N-heterocyclic carbenes, to raise the energy of the metal e_g_ orbitals or strong π-acceptor ligands to lower the energy of the metal t_2g_ orbitals.^36^ To increase the bond covalence between the metal and the ligand, π-donor ligands, such as amido ligand, can be used.^37^ This is a particularly effective strategy for extending MLCT excited-state lifetimes of d^6^ metal complexes by destabilizing their MC states, a process known as “HOMO inversion”.^38^ Additional strategies include optimizing the bite angle of chelating ligands to increase the metal–ligand orbital overlap and thereby increasing the ligand field strength or employing structurally rigid ligands to minimize excited-state distortion.^39,40^

Another successfully employed strategy for implementing base metal complexes in photocatalysis involves a mechanistic switch from outer- to inner-sphere reactivity. This approach leverages ligand-to-metal charge transfer (LMCT) states, which are the result of an electronic transition from a filled orbital that is mainly based on a ligand to an empty orbital of the metal center (Scheme 1C).^35,41^ Since the empty orbital on the metal center must be low in energy to be accessible, LMCT states are characteristic of complexes that bear electrophilic, high valent metal centers, such as Cu^II^, Fe^III^, and Co^III^. Conversely, since the ligand becomes oxidized during this transition, electron-rich σ- or (σ + π)-donor ligands, such as carboxylates, alkoxides, halides, or azides, generally favor LMCT. These LMCT excited states can generally be accessed upon visible-light irradiation and can potentially trigger the homolysis of the metal–ligand bond to generate reactive radical intermediates. The key advantage of this approach is that it bypasses the need for long-lived excited states by precoordinating with the nucleophilic substrate, allowing LMCT states with very short lifetimes to still engage in efficient reactions.^42^

In the upcoming sections of this review, we will highlight recent key advances in the implementation of base metal photocatalysts in photoredox-catalyzed transformations. We will be centered on examples employing copper, iron, chromium, cobalt, manganese, and vanadium catalysts. This tutorial review will be limited to examples in which the base metal is the key light absorbing species in the photocatalytic cycle. Discussions of dual catalytic examples in which the base metal,^13^ often nickel,^43–46^ is not the primary light absorbing species will not be included, as these advances have already been elegantly described in prior reviews.

## 2 Recent Examples Leveraging Base Metal Photocatalysts

### 2.1 Copper

Copper has rapidly emerged as one of the most versatile and widely studied base metals in photoredox catalysis.^47–50^ Because of its higher natural abundance, cost-efficiency, and intrinsic photoluminescence, copper-based visible-light photoredox catalysis stands out as a sustainable and affordable alternative to costly noble metal catalysts like ruthenium and iridium. Homoleptic and heteroleptic copper complexes have multiple readily accessible oxidation states, Cu^I^, Cu^II^, and Cu^III^, enabling the design of catalytic cycles for both substrate activation and bond construction through radical pathways under low-energy visible light. Copper complexes offer several advantages for photocatalysis including the possibility for fine-tuning of the absorption and emission properties, long excited-state lifetimes, and a characteristic structural flexibility.^51^ This tutorial review will highlight recent advances in copper-based photocatalysis, focusing on representative studies that showcase the unique reactivity and versatility of copper complexes in visible-light-mediated transformations.

Huang’s group recently reported a copper-photoredox strategy that achieves selective defluorinative C–O coupling between trifluoromethylarenes and alcohols through a dual catalytic system (Scheme 2A), motivated by the challenge of achieving direct and selective C–F bond activation using base metal photoredox catalysis.^52^ This reaction proceeds via an MLCT-excited Cu^I^ species and operates through an outer-sphere electron-transfer mechanism under visible-light irradiation. The transformation operates via two interlinked cycles: C–F activation and C–O bond formation (Scheme 2B). In the presence of LiI and Zn­(OAc)_2_, the Cu^I^ precursor ligated by 1,2-bis­(diphenylphosphino)­benzene (dppbz) forms a homoleptic bisphosphine complex (**CuP**_**4**_), which serves as the photocatalyst. Upon excitation, *CuP_**4**_ reduces the trifluoromethylarene (**1**), generating an aryl difluoromethyl radical (**2**) through mesolytic C–F cleavage while simultaneously producing an oxidized Cu^II^ species (**3**). Parallel to this, a lower-coordinate Cu^I^–dppbz complex (**CuP**_**2**_) engages in the C–O coupling cycle, forming Cu^I^–alkoxide intermediate (**4**) under visible-light irradiation in the presence of Zn­(OAc)_2_. The two cycles converge when **3** and **4** undergo electron transfer to form Cu^II^–alkoxide **5** while regenerating **CuP**_**4**_. **2** and **5** can then react with each other to deliver the difluorobenzyl ether product (**6**). LiI and Zn­(OAc)_2_ are essential for stabilizing **CuP**_**4**_, promoting C–F bond cleavage by fluoride capture, and balancing the two cycles. Notably, interception of **2** by iodide generates Ar–CF_2_I (**7**), which acts as a radical reservoir, releasing **2** back into the cycle under photoredox conditions. This dual copper catalysis not only overcomes the challenge of strong C–F activation but also broadens the reaction scope to aliphatic alcohols, a significant advance compared to prior methods limited to phenolic substrates or radical-trapping reagents.^53,54^

**Scheme 2:**
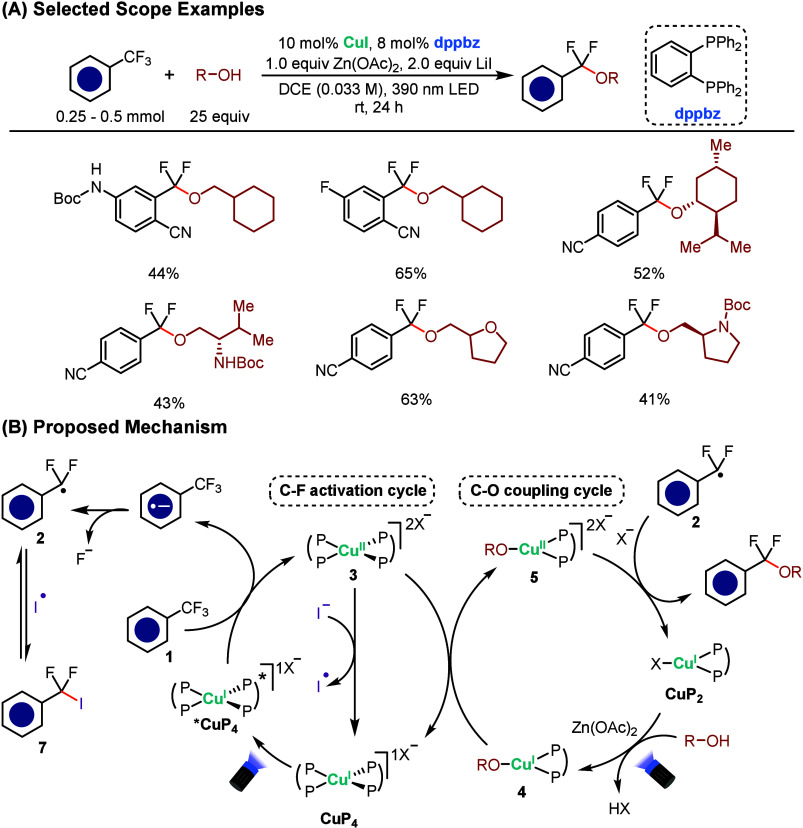
Copper-Catalyzed Defluorinative C–O Coupling of Trifluoromethylarenes and Alcohols

Guo and Zhang’s group recently reported a photoinduced copper-catalyzed three-component asymmetric alkylalkynylation of alkenes via an intermolecular hydrogen atom transfer (HAT) strategy (Scheme 3).^55^ Building on their earlier work involving intramolecular radical translocation in cyclic amines,^56^ they extended the approach to a broader set of C­(sp^3^)–H-containing feedstocks. The main key to the transformation is the use of modified aryl halides as HAT reagents (**8** and **9**), which following reduction by an excited *Cu^I^–alkyne intermediate to generate aryl radicals that are capable of selectively abstracting protic or hydridic C­(sp^3^)–H bonds to form alkyl radicals. The resulting alkyl radicals then participate in copper-catalyzed asymmetric radical cross-coupling with terminal alkynes and alkenes, enabling the formation of enantioenriched alkyne derivatives. This reaction proceeds through visible-light excitation of a Cu^I^ complex to an MLCT state, followed by inner-sphere reactivity. Notably, copper catalysis plays a central role, enabling enantioselective control through coordination of cinchona alkaloid-derived ligand **10** and facilitating previously inaccessible radical coupling pathways, including reactions with substrates like acetonitrile, ketones, esters, ethers, and amides. This work underscores the potential of copper-based photoredox catalysis for selective C–H activation and complex molecule construction, particularly in synthesizing diverse chiral aliphatic alkynes with high chemo-, regio-, and enantioselectivity.

**Scheme 3:**
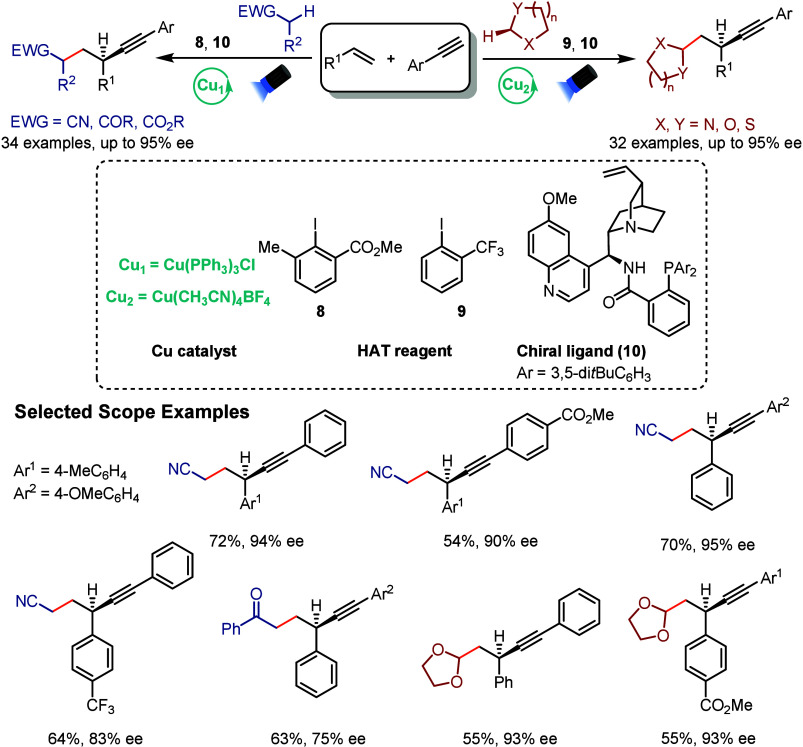
Copper-Photocatalyzed Enantioselective Alkylalkynylation of Alkenes

Ritter’s group recently developed a photoactive Cu^I^ catalyst supported by an electron-rich BINAP-type ligand that enables Kharasch-type haloalkylation of alkenes under visible-light irradiation (Scheme 4).^57^ The study is motivated by the limitations of classical Kharasch additions and leverages copper photocatalysis with redox-active esters to enable the mild haloalkylation of alkenes, providing access to highly functionalized alkyl groups and synthetically versatile α-halo carbonyl products. Mechanistic studies show that the Cu^I^–BINAP complex performs two distinct roles: upon blue-light excitation, the MLCT-excited Cu^I^ species serves as a photocatalyst to generate alkyl radicals from redox-active esters (RAEs), and it subsequently acts as a halogen atom transfer (XAT) agent to trap the carbon-centered radicals with a halogen, forming new C–C and C–X bonds in a single step. By uniting photoredox and XAT roles in one catalyst, the system bypasses the need for separate precious metal photoredox catalysts or radical initiators used in traditional Kharasch additions, providing a highly efficient route for the difunctionalization of alkenes. Notably, the BINAP-derived ligand was crucial for achieving high photocatalytic activity, likely because of their strong electron-donating nature which was found to raise the Cu^I^/Cu^II^ redox potential while also extending the excited-state lifetime of the copper complex.

**Scheme 4:**
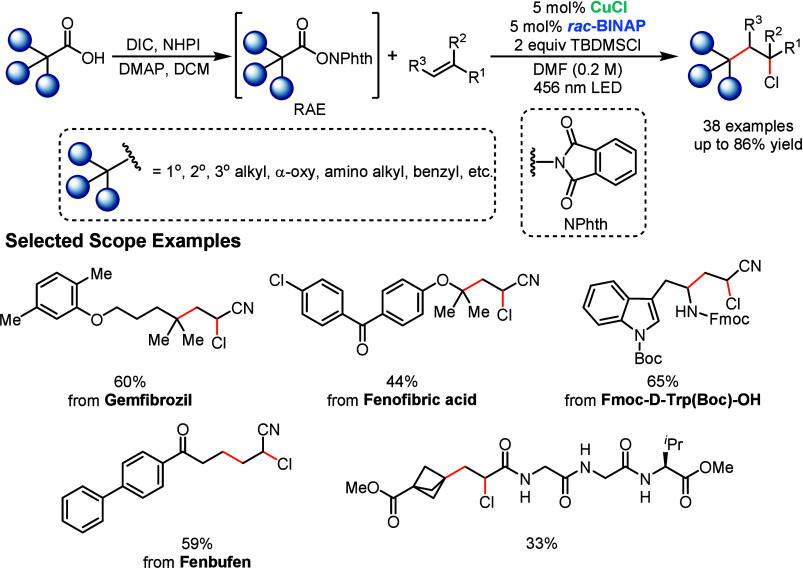
Copper-Photocatalyzed Kharasch-Type Haloalkylation of Alkenes

Motivated by the need to overcome the poor coordinative stability and mechanistic ambiguity of existing phenanthroline-based Cu^I^ photoredox catalysts, Bissember and co-workers recently reported a series of Cu^I^ photoredox catalysts featuring tetradentate phenanthroline ligands with tethered donors (phosphine, phosphinite, phosphite, oxazoline, pyrazole).^58^ These complexes addressed the instability of earlier bidentate systems while maintaining desirable photophysical and redox properties. Among these, copper complex **11** bearing a tetradentate, phenanthroline-type ligand with tethered dicyclohexylphosphinite groups emerged as the standout: while **11** was slightly less effective than their benchmark copper photocatalyst, [Cu­(bcp)­(POP)]­PF_6_ (**12**), in aryl halide hydrodehalogenation (Scheme 5A), **11** mediated the trifluoromethylchlorosulfonylation of alkenes more effectively than **12** (Scheme 5B). Similar to other Cu^I^ photocatalysts, the reaction proceeds through an outer-sphere mechanism from an MLCT excited state. The photocatalytic activity of phenanthroline-based Cu^I^ complexes was found to depend on finding an optimal balance of ligand rigidity and flexibility, enabling both stabilization of the metal center and dynamic reorganization upon photoactivation. This coordinative metastability proved key to the catalytic activity of these Cu^I^ complexes, representing a deviation from traditional design principles for metal-based photocatalysts that prioritize high quantum yields and long emissive lifetimes. Notably, complex **11** demonstrates high catalytic performance despite weak luminescence, highlighting that photophysical properties alone do not solely dictate catalytic efficacy. This finding contrasts with prevalent design strategies for first-row photoredox catalysts which emphasize highly luminescent complexes with extended excited-state lifetimes to enable efficient photochemistry.^32^ The activity of complex **11** demonstrates that strong emission is not a requirement for photocatalytic competence and that weakly emissive excited states can still drive productive photoredox processes when electron-transfer pathways are kinetically favorable.

**Scheme 5:**
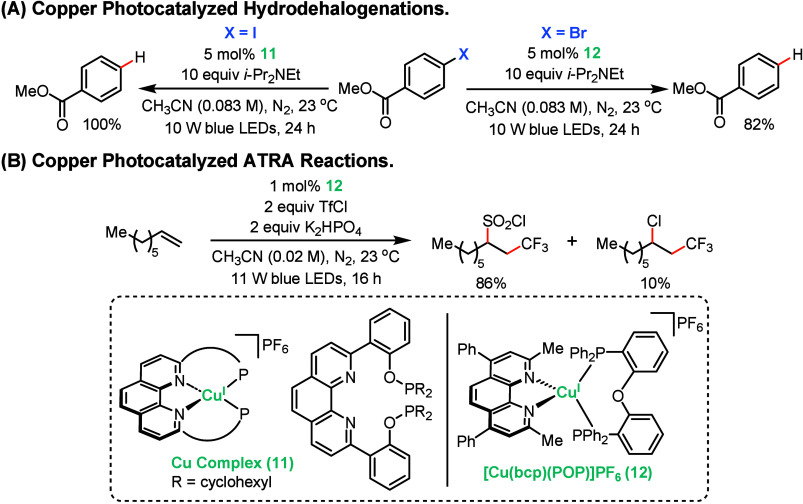
Copper-Photocatalyzed Hydrodehalogenation and Atom-Transfer Radical Addition Reactions

In 2024, Abderrazak and Reiser reported a copper-photocatalyzed strategy for the divergent synthesis of organic thiocyanates and isothiocyanates using benzylic thiocyanates as atom-transfer radical addition (ATRA) reagents (Scheme 6A).^59^ This study was motivated by the desire to broaden copper-photocatalyzed ATRA chemistry to achieve selective access to both thiocyanate and isothiocyanate products. The transformation proceeds via MLCT-excited Cu^I^ and LMCT of a Cu–NCS species, encompassing both inner- and outer-sphere mechanisms. Upon green (530 nm) LED irradiation, the Cu^I^ phenanthroline complex Cu­(dap)_2_Cl reduces the benzylic thiocyanate to generate a benzylic radical and a Cu^II^–thiocyanate intermediate. The benzylic radical subsequently adds to an alkene, and the product outcome is dictated by the electronic properties of the substrate: electron-deficient styrenes selectively formed thiocyanates (C–S bond formation), whereas electron-rich styrenes gave isothiocyanates (C–N bond formation). Substrates with neutral electronics afforded mixtures, but the selectivity could be tuned by adding exogenous thiocyanate salts.

**Scheme 6:**
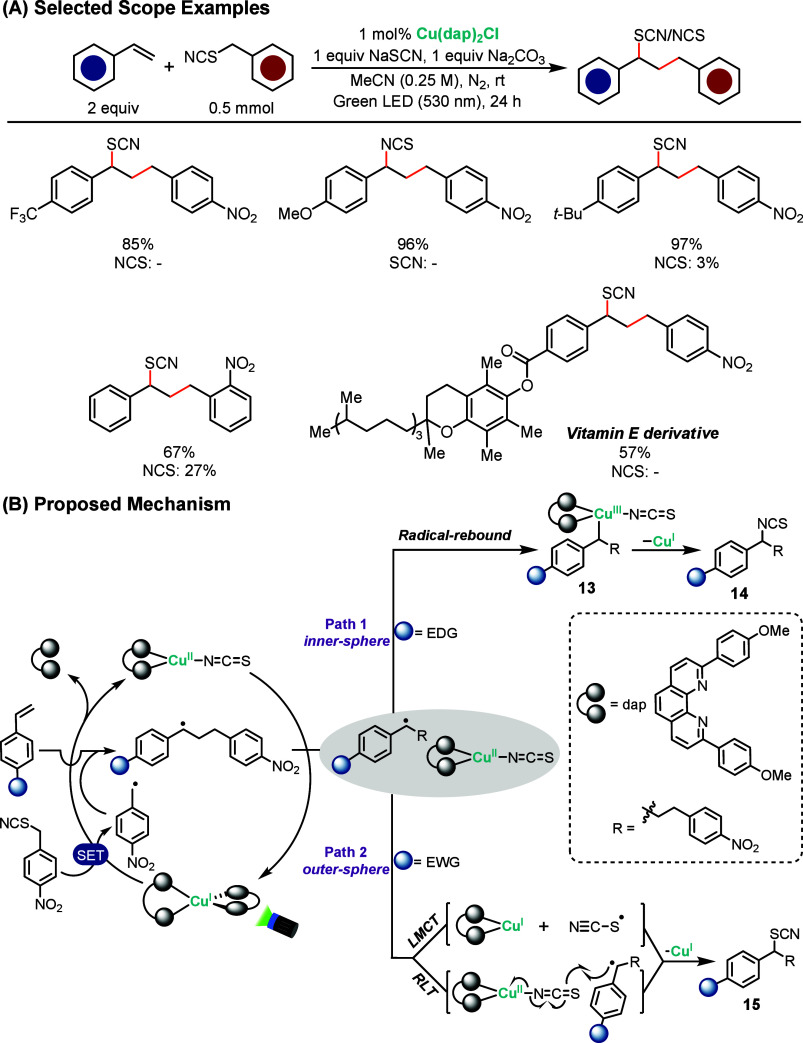
Copper-Photocatalyzed Divergent Access to Organic Thio- and Isothiocyanates

Mechanistic studies showed that the copper catalyst operates through two pathways depending on the radical polarity (Scheme 6B). Electron-rich radicals undergo inner-sphere coordination with Cu^II^, leading to transient Cu^III^ complexes (**13**) that undergo reductive elimination to form C–N bonds and yield isothiocyanates (**14**). Conversely, electron-poor radicals react in an outer-sphere manner, either through radical–ligand transfer or capture of a free ^•^SCN radical, to produce thiocyanates (**15**). This work highlights the unique ability of copper complexes to control divergent bond construction, enabling both chemo- and regioselective access to valuable thiocyanate and isothiocyanate motifs with broad synthetic utility.

In 2018, Rehbein and Reiser reported a visible-light-accelerated Cu^II^-catalyzed oxoazidation of vinyl arenes that enables the regio- and chemoselective formation of α-azidoketones (**16**) from simple alkenes (**17**) using trimethylsilyl azide (**18**) and molecular oxygen under mild conditions (Scheme 7A).^60^ The motivation behind this work was to develop a catalytic and step-economical alternative to existing azidoketonization methods that rely on stoichiometric oxidants or prefunctionalized substrates. Using this approach, electron-withdrawing aryl substrates (**19** and **20**) were successfully converted to the corresponding α-azidoketones in moderate to good yields, while heteroaryl vinyl substrates such as thiophene and benzofuran derivatives (**21** and **22**) were also well-tolerated, highlighting the broad functional-group compatibility of the Cu-catalyzed oxoazidation under visible-light conditions.

**Scheme 7:**
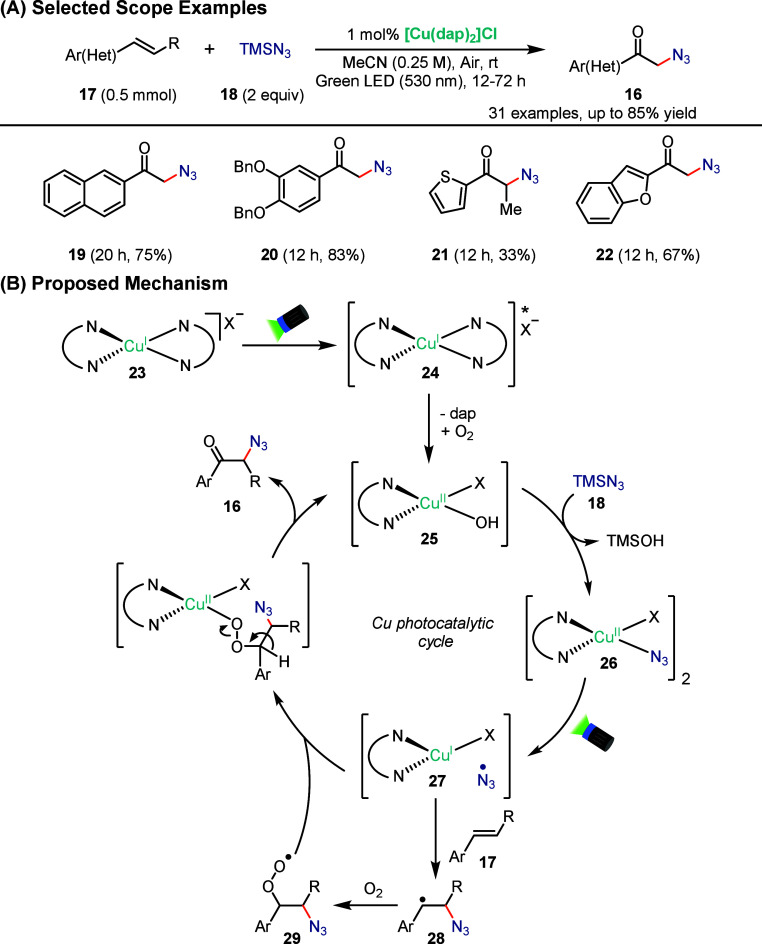
Copper-Photocatalyzed Regio- and Chemoselective Oxoazidation of Vinyl Arenes

The reaction is uniquely promoted by phenanthroline-ligated copper complexes (**23**), with Cu^II^ identified as the catalytically active species (Scheme 7B). Mechanistically, aerobic oxidation of the Cu^I^ precatalyst (**23**) generates a Cu^II^–dap complex (**24**) that coordinates the azide to form an azide-bridged Cu^II^ species (**26**) that absorbs in the visible region (530 nm). Visible-light excitation triggers homolytic cleavage of the Cu^II^–N_3_ bond through LMCT, producing a Cu^I^ species and an azide radical (**27**), which adds regioselectively to the styrene (**17**) to form a stabilized benzylic radical (**28**). Subsequent trapping by O_2_ generates an O-centered radical (**29**) that undergoes inner-sphere radical rebound at copper, forming the C–O bond and releasing the α-azidoketone (**18**) while regenerating the Cu^II^ catalyst (**25**), closing the catalytic cycle.

In 2016, Peters and Fu reported the first copper-photocatalyzed enantioselective cross-coupling between arylamines and tertiary alkyl chloride electrophiles.^61^ Motivated by the importance of these scaffolds in bioactive molecules, they continue to develop diverse methodologies, broadening the scope of the transformation. In a recent example from 2022, they reported the cross-coupling between racemic tertiary electrophiles and aniline derivatives.^62^ The reaction is photocatalyzed by CuCl in combination with chiral SEGPHOS ligand (*R*)-**30** and is an example of an inner-sphere LMCT mechanism. Selected examples from the reaction scope are shown in Scheme 8A. Tertiary alkyl chlorides bearing an electron-withdrawing cyano group were well-tolerated, providing the desired cross-coupled products in good yield and high *ee* (**31**–**35**). Notably, they were able to tolerate pendant olefins, providing **32** in 71% and 92% *ee* with no observed side reactivity. For substrates bearing two C–Cl bonds, the reaction was selective for the more electron deficient site, providing **33** in 78% yield and 91% *ee*. Heterocyclic aniline substrates also reacted smoothly, including a dibenzofuran analog (**35**). Finally, the reaction was also compatible for tertiary alkyl chlorides bearing electron-withdrawing amide groups, providing access to disubstituted unnatural amino acid scaffolds (**36**, **37**).

**Scheme 8:**
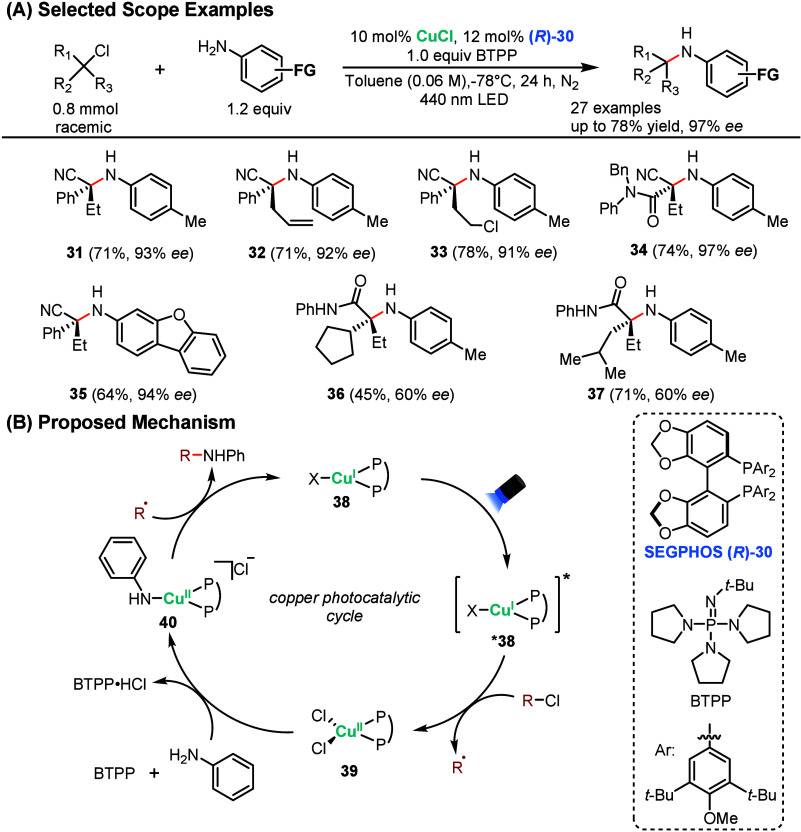
Copper-Photocatalyzed Enantioselective Alkylation of Anilines

The proposed mechanism is outlined in Scheme 8B. Upon 440 nm irradiation, the excited CuCl–SEGPHOS complex *38 undergoes a chlorine atom-transfer reaction to generate R^•^ and complex **39**. Complex **39** then undergoes base-induced substitution by the aniline to furnish copper complex **40**, which combines with R^•^ to provide the cross-coupled product while regenerating the Cu^I^ complex **38**. This mechanistic proposal was supported by EPR measurement, UV–vis analysis, cyclic voltammetry, and Stern–Volmer analysis.

### 2.2 Iron

Iron is a highly abundant, cheap, and nontoxic metal, with many complexes that are stable under ambient conditions, making iron a natural candidate for photocatalyst development. Because of these advantages, there has been increased interest in recent years in the development of iron photocatalyzed transformations.^63,64^ Several strategies for leveraging iron in photocatalysis have emerged, which include photoinduced outer-sphere and inner-sphere electron-transfer mechanisms involving both metal-centered and charge-transfer states. This includes LMCT, where the metal center is reduced and the corresponding ligand oxidized after electronic excitation, typically with visible-light irradiation. This is an inner-sphere mechanism by nature. These LMCT states have recently been extensively exploited with iron to promote decarboxylation of nonaromatic carboxylic acids, hydroazidation of alkenes from cyanide salts,^65^ and the generation of halogen radicals for C–H functionalization.^35^

Taking advantage of this concept, Juliá-Hernández and co-workers recently reported an iron photocatalyzed trifluoromethylation of aromatic cycles using trifluoracetic acetate as the fluoroalkane source (Scheme 9A).^66^ Trifluoromethylated compounds are key components of industrial chemistry nowadays. Generally, decarboxylation of trifluoromethyl acetate (**41**) to generate trifluoromethyl radicals requires strongly oxidizing conditions, limiting its application as a reagent in synthesis. To circumvent this, the authors implemented an LMCT strategy using Fe­(OTf)_2_ as the catalyst in combination with ligand **42**, switching radical formation from **42** to a more accessible inner-sphere mechanism. It is worth noting that in absence of **42**, the reaction yield drops significantly, as the ligand was found to be crucial in inducing the bathochromic shift necessary for efficient visible-light absorption. The reaction was found to be compatible with a range of electron rich nitrogen-based heterocycles (**43** and **44**), coumarins (**45**) and a protected uridine derivative **46**, with the latter examples representing potentially interesting bioactive compounds.^67^ Lastly, their trifluoromethylation protocol was also effective for ferrocene, yielding **47** in 24% yield.

**Scheme 9:**
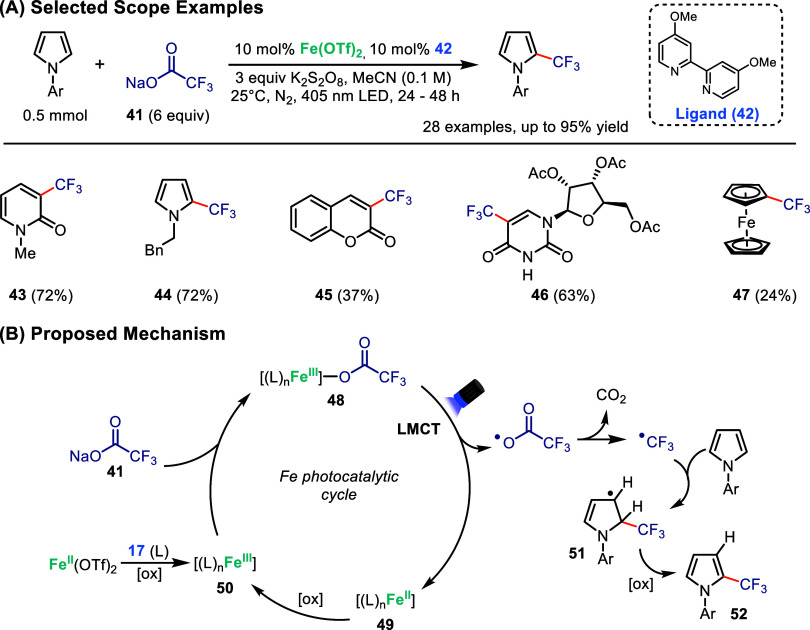
Iron-Photocatalyzed Trifluoromethylation of Arenes

Mechanistically, this work represents a typical application of LMCT (Scheme 9B). In the first steps, the active iron catalyst **50** couples with **41** through ligand exchange to generate intermediate **48**. Under visible-light irradiation, **48** then undergoes LMCT to yield the reduced iron complex **49**, and the oxidized acetate undergoes decarboxylation to generate a trifluoromethyl radical that can be trapped by an electron-rich heterocycle to generate intermediate **50**. Oxidation of **51** yields the final fluoroalkylated product (**52**). The mechanism was supported by UV–vis analysis and radical trapping experiments.

Over the years, the West group has reported several transformations leveraging iron LMCT photocatalysis, including azidation and decarboxylative protonation reactions.^68–70^ Building on this prior work, in 2023 the West group employed iron LMCT photocatalysis for the hydrofluoroalkylation of unactive alkenes under visible-light irradiation using di- and trifluoroacetic acid as inexpensive sources of ^•^CHF_2_ and ^•^CF_3_ radicals, respectively (Scheme 10).^71^ This transformation was made possible by a polarity-match between the fluoroalkyl radical and the unactivated alkene, facilitating selective radical addition, and by using catalytic TRIP thiol (or the corresponding disulfide) as a HAT reagent to effectively terminate the reaction. A broad substrate scope with yields ranging from moderate to excellent, mild redox-neutral conditions and the potential for late-stage functionalization were showcased in these transformations, with selected examples highlighted in Scheme 10. Product **53** demonstrates the chemoselectivity toward unactivated alkenes, which was formed in 81% yield as a single isomer. Selective decarboxylation of trifluoroacetic acid in the presence of unactivated carboxylic acids was observed (**54**), and nitrogen protecting groups like phthalimide were well-tolerated (**55**). Oxetane-containing derivatives were also effective substrates, yielding compound **56** in 73% yield. A broad range of bioactive molecules, including vinclozolin (**57**), a potent fungicide, and pregnenolone (**58**), an important steroid, also underwent efficient fluoroalkylation, highlighting the method’s potential for late-stage functionalization.

**Scheme 10:**
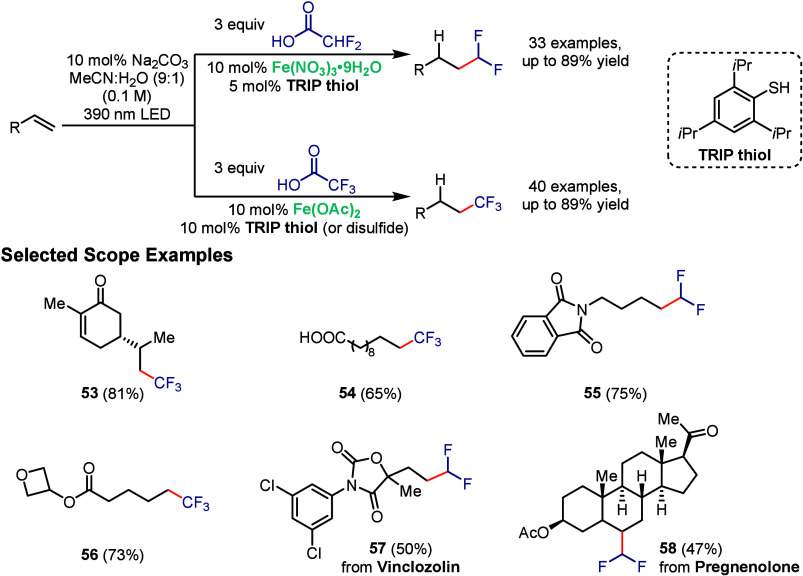
Iron-Photocatalyzed Hydrotrifluoroalkylation of Unactivated Alkenes

As previously discussed, iron can, through LMCT, generate radicals from fluorinated carboxylic acids; however, having fluorine atoms is not a necessity to access this reactivity. In 2024, Ackerman-Biegasiewicz and co-workers reported an iron photocatalyzed coupling between alkenes and carboxylic acids (Scheme 11A).^72^ In this work, adding “dien”, a simple ligand, enabled previously unsuccessful transformations under prior decarboxylative Giese reactions by generating a more photoactive intermediate. While the metal catalyst itself is an important aspect of reaction sustainability, their use of acetonitrile, considered a green solvent, and of alkenes and carboxylic acids as starting materials, substrates that are part of large chemical feedstocks, further emphasizes the sustainability of the transformation. With their decarboxylative Giese method, they coupled a broad scope of carboxylic acids and activated alkenes. For example, ester **59** was obtained with 90% yield on a gram scale, showing the effectiveness of the transformation on larger scale, which could lead to faster industrialization. Brominated adamantine **60** was also compatible, with no competitive XAT being observed, and amino acids could be employed directly as radical precursors (**61**). Finally, a derivative of osimertinib, a tyrosine kinase inhibitor, was functionalized in 58% yield (**62**), highlighting the possibility for late-stage functionalization.

**Scheme 11:**
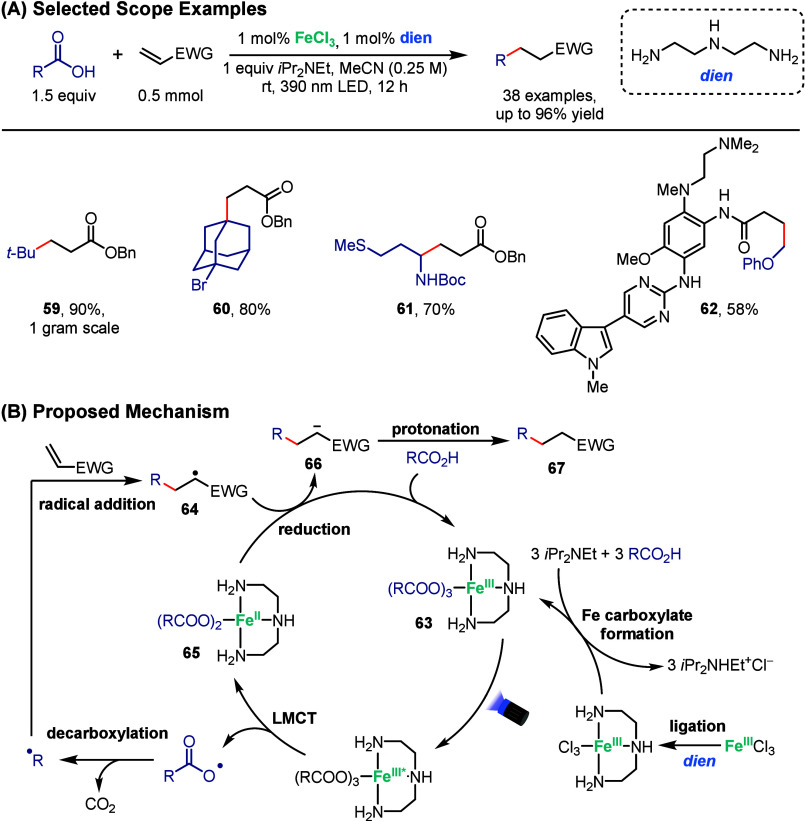
Iron-Photocatalyzed Decarboxylative Giese additions

The mechanism of the transformation is initiated in a similar manner to those previously described, where the carboxylic acid is deprotonated and complexes with iron to form iron carboxylate species **63** (Scheme 11B). Excitation and oxidative LMCT lead to decarboxylation, generating an alkyl radical that subsequently reacts with the activated alkene to generate intermediate **64**. SET between iron complex **65** and intermediate **64** leads to anion **66**, which after protonation generates the final Giese adduct (**67**). An interesting point to note is that the transformation is redox-neutral and does not require any additional oxidants. Additional DFT and UV–vis studies provided support for complex **63** being the photoactive species. Radical trap experiments were conducted to provide support for the proposed mechanism.

In 2021, the Rovis group reported a unique method for C­(sp^3^)–H alkylation reactions using FeCl_3_ as the photocatalyst for the preparation of valuable substituted ketone products (Scheme 12).^73^ Upon 390 nm irradiation, photoinduced LMCT at the iron center generates a chlorine radical, which can undergo HAT with C­(sp^3^)–H bonds at the β-carbon of aliphatic ketones to generate the corresponding alkyl radical. These alkyl radicals can undergo standard Giese type reactions (product **a**), or, at increased temperatures, can undergo a 1,2-migration prior to the Giese addition step to generate rearranged-alkylated products (product **b**). While the 1,2-migration products have a higher apparent activation energy, the resulting tertiary radical is more stable, and consequently, under thermodynamic control, the rearranged products are favored. With this method, they generated **68** in 59% yield and an **a**:**b** ratio of 1:1.4 at room temperature (Scheme 12, Conditions A), while at 60 °C (Conditions B) they observed a 64% yield and an **a**:**b** ratio of 1:10. When the ketone is replaced by *p*-OAc-phenyl (**69**), the **a**:**b** ratio becomes 20:1 using Conditions A and 1:1 using Conditions B. This can be explained by the unfavored formation of the cyclic intermediate that would break aromaticity, therefore favoring the unrearranged product. Finally, product **70** was formed with reversed selectivity, favoring the rearranged form. The authors rationalized these observations using two factors: the Thorpe–Ingold effect, with high steric hindrance favoring the cyclization after 1,2-migration, and the formation of a more stable and nucleophilic tertiary radical. Cyclization itself is made possible by the bis­(cyano)-stabilized anion intermediate.

**Scheme 12:**
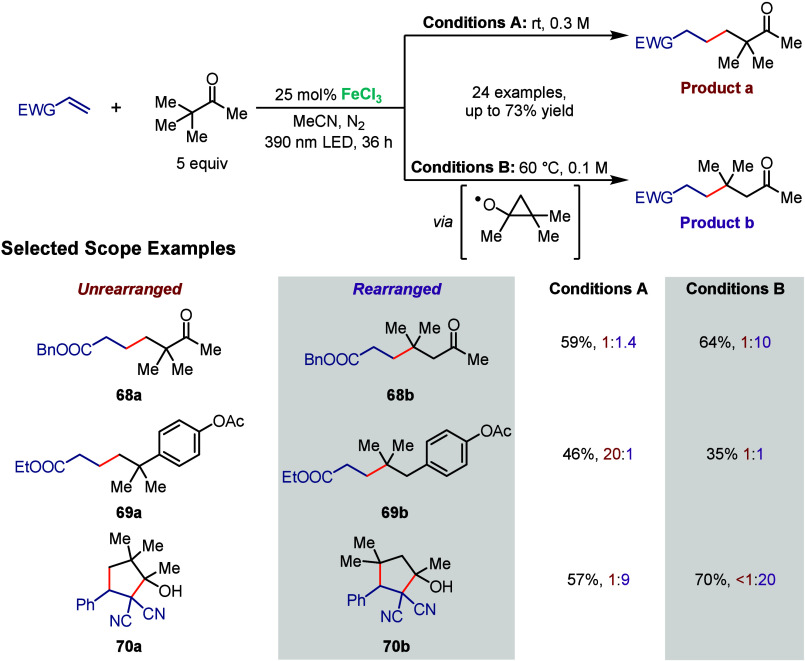
Iron-Photocatalyzed C­(sp^3^)–H Alkylations

In 2022, Xue and co-workers reported the β-scission of unstrained cyclic alcohols followed by amination with di-*tert*-butyl azodicarboxylate (DBAD) using Fe­(acac)_3_ and sodium *tert*-butoxide (*tert*-BuONa) as a cocatalyst (Scheme 13A).^74^ This kind of chemistry is usually limited to cerium, making this method an attractive alternative because of the abundance and affordability of iron.^75^ By applying their optimal conditions, they reached 93% yield of the aminated product starting from cyclohexanol **71**. Alkynes were also tolerated, albeit in decreased yields (**72**). Tertiary gave excellent results, yielding the aminated product **73** in 84%. Cyclohexanol with a potentially chelating pyridine was also tolerated, yielding to **74** with 42%. Cyclobutanol and cyclooctanol were also converted to the corresponding aminated products in 79% and 27% yield, **75** and **76** respectively. Interestingly, **77** was obtained in good yield (91%), showing that the geminal substituent does not need to be an sp^2^-carbon for the reaction to proceed. Their proposed mechanism was supported by UV–vis and initial rate measurements.

**Scheme 13:**
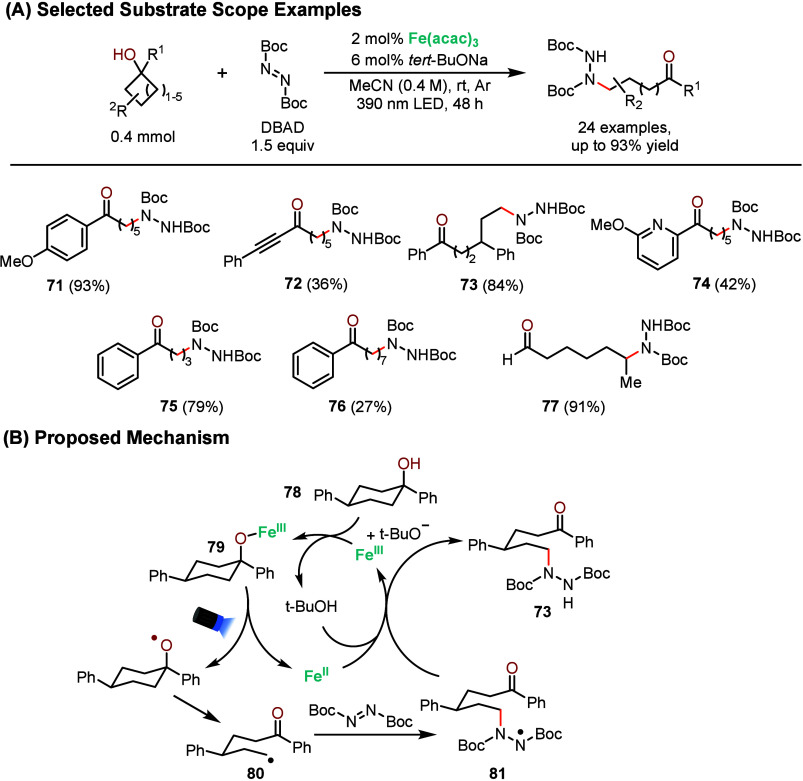
Iron-Photocatalyzed C–C Bond Cleavage and Amination of Cyclic Alcohols

The proposed mechanism starts with the deprotonation of the cyclic alcohol **78** followed by coordination with the iron center (Scheme 13B). Under light, the intermediate **79** is transformed into alkoxy radical by LMCT, follow by β-scission to generate radical intermediate **80** which can be trapped by DBAD to form **81**. After SET with iron and protonation by *tert*-BuOH, the aminated product **73** is obtained and the two catalysts are regenerated. This mechanism is another example of a net redox-neutral catalytic cycle. The authors performed a series of mechanistic studies, such as UV–vis measurements and radical trap experiments, to provide support for their proposed mechanism.

### 2.3 Cobalt

Recently, cobalt complexes have garnered increased attention as sustainable replacements for precious metal systems in photocatalysis. Under photochemical conditions, cobalt can access multiple oxidation states, from Co^0^ to Co^III^, allowing for them to engage in both one- and two-electron-mediated bond-formation reactions throughout photocatalysis or in combination with photoredox catalysis.^76,77^ This section will highlight recent applications of cobalt complexes in visible-light-mediated transformations, with a focus on studies that showcase their unique excited-state reactivity.

In 2023, MacMillan, McCusker, and co-workers reported their progress in solving the enduring challenge of shorter lifetimes of the excited-states of first-row transition metal complexes.^78^ Although first-row base metals offer numerous sustainability benefits, their application in photocatalysis remains constrained because of their relatively small ligand-field energy gaps, which accelerate nonradiative decay and consequently reduce their effectiveness. In this study, the authors demonstrated that Co^III^ polypyridyl complexes could be used as photocatalysts by exploiting Marcus inverted region behavior that couples increases in excited-state energies with increased excited-state lifetimes. The authors demonstrated that [Co­(4,4′-Br_2_bpy)_3_]^3+^ (**82**) showed strong redox potentials and a sufficiently long excited-state lifetime to effectively photocatalyze C­(sp^2^)–N couplings of aryl amides with arylboronic acids (Scheme 14A). Notably, **82** exhibited superior outer-sphere reactivity in comparison to the conventional precious metal iridium and ruthenium photocatalysts as well as the common organic dye 4CzIPN, all of which were generally unsuccessful at promoting the same coupling reactions. Mechanistic studies, including radical clock studies and Stern–Volmer quenching, revealed the generation of amidyl radical intermediates through direct oxidation of the aryl amide by the excited *Co^III^ species, which then undergoes a metal-free *ipso*-substitution with the arylboronic acid (Scheme 14B), bypassing substrate limitations of traditional Chan–Evans–Lam couplings. The reported methodology showed broad substrate scope, accommodating electron-rich and electron-poor substrates, halogenated arenes, and even *ortho*-substituted boronic acids, all under mild conditions. This work established a new paradigm for first-row transition-metal photocatalysis, demonstrating that cobalt complexes can overcome intrinsically short ligand-field excited-state lifetimes by operating in the Marcus inverted region, thereby enabling direct visible-light generation of N-centered radicals from simple amides.

**Scheme 14:**
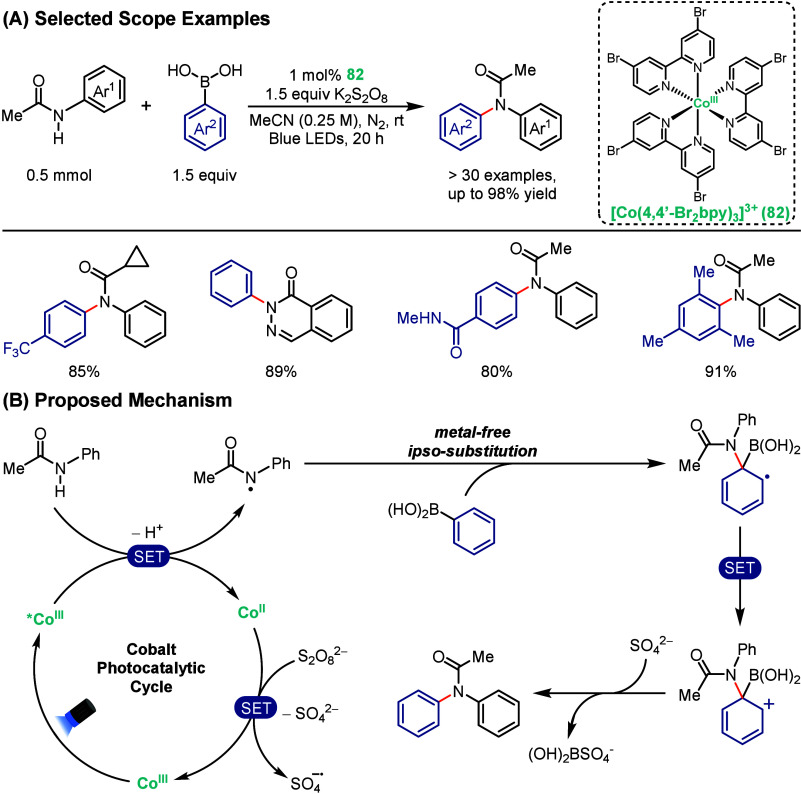
Cobalt-Photocatalyzed C­(sp^2^)–N Coupling Reaction between Aryl Amides and Arylboronic Acids

In a complementary strategy, Wolf and co-workers demonstrated that efficient photocatalysis can proceed even with an ultrashort-lived excited-state.^79^ In this study, to perform direct C–H arylation of pyrroles with aryl halides, a bis­(diiminopyridine) cobalt complex, **83**, was employed under visible-light irradiation. Despite an excited-state lifetime of only ∼8 ps, the photocatalyst promoted reductive couplings with electron-deficient aryl halides in moderate to good yields (Scheme 15A). While electron-deficient aryl bromides gave higher reaction yield, electron-donating substrates showed lower reactivity. Notably, these results are comparable to the catalytic reactivity of conventional organic dyes, such as 4CzIPN and rhodamine 6G. Mechanistic investigations revealed a consecutive photoinduced electron transfer (conPET) process, which is more commonly observed with organic dyes. UV/vis/NIR spectroscopy, ^1^H NMR spectroscopy, and DFT studies, supported the formation of a weak ground-state outer-sphere donor–acceptor complex between **83** and NEt_3_, enabling the rapid formation of the one-electron reduced **83** ^**I**^ species under visible-light irradiation (Scheme 15B). Upon excitation, **83** ^**I**^ undergoes SET with the aryl halide, leading to reductive C–X bond cleavage, which subsequently couples with *N*-methylpyrrole through a radical chain mechanism similar to that proposed by Lenori and co-workers.^80^ This preorganization strategy bypasses diffusion-controlled kinetics and illustrates how cobalt photocatalysts can operate effectively without the need for microsecond lifetimes. Collectively, these findings highlight that inner-sphere, LMCT-driven cobalt photocatalysis can promote synthetically useful transformations even in the absence of long-lived excited states.

**Scheme 15:**
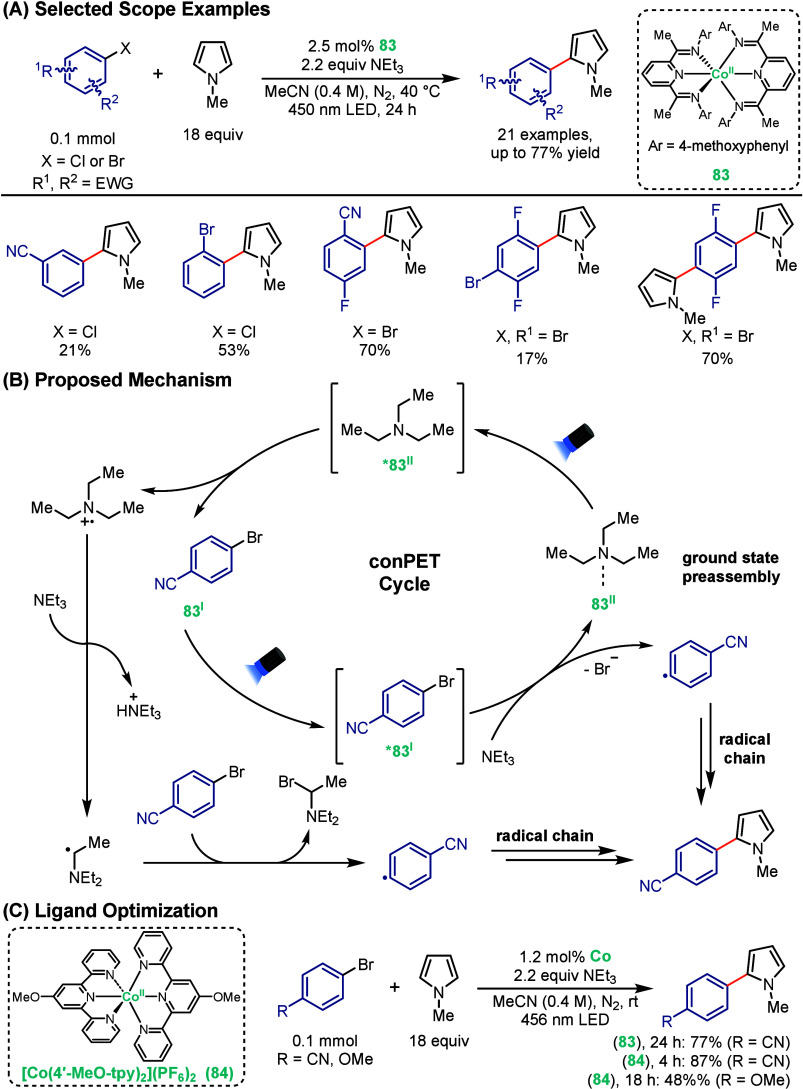
Cobalt-Photocatalyzed C–H Arylation of Pyrroles

Building on the earlier conPET-based strategy, Wolf’s group has improved their catalyst design by replacing the diiminopyridine ligand with terpyridine (tpy) to yield [Co­(4′-MeO-tpy)_2_]­(PF_6_)_2_ (**84**) for photochemical arylation reactions (Scheme 15C).^81^ While their previous bis­(diiminopyridine) cobalt complex **83** demonstrated efficient catalysis is achievable despite ultrashort excited-state lifetimes, the terpyridine scaffold provided superior reactivity and broader substrate scope. In particular, the system showed improved tolerance for electron-donating aryl halides and accelerated reaction rates, achieving high yields under mild conditions. Mechanistically, alongside the conPET pathway, α-aminyl radical-mediated halogen atom transfer (XAT) was also found to contribute to substrate activation. Similar to the author’s prior work, complex **84** also demonstrates inner-sphere, LMCT-driven photocatalysis, in addition to better tolerance for electron-donating aryl halides.

While the prior examples showcased cobalt photocatalysts involved in excited-state SET reactions, macrocyclic tetradentate cobalt complexes, such as vitamin B_12_, have been shown to mediate radical formation under visible-light irradiation through a unique mechanism, proceeding through LMCT excitation of alkylated cobalt intermediates.^82,83^ In a recent example, Pitre and co-workers reported a method for the cyclopropanation of electron-deficient alkenes using dichloromethane (CH_2_Cl_2_) as the methylene source using vitamin B_12_ photocatalysis (Scheme 16).^84^ By leveraging the highly nucleophilic Co^I^ oxidation state of vitamin B_12_, CH_2_Cl_2_ undergoes S_N_2-type nucleophilic substitution to form a Co^III^–CH_2_Cl intermediate (**85**), which can be photolyzed with green (525 nm) LED irradiation to generate a ^•^CH_2_Cl radical. This radical participates in a polarity-matched Giese addition to electron-deficient alkenes, followed by trapping of the carbon-centered radical by Co^II^ to yield intermediate **86**. Finally, reduction of **86** followed by 3-*exo*-*tet* cyclization delivers the desired cyclopropane product. UV–vis studies provided support for the proposed S_N_2 mechanism, while radical trapping experiments provided evidence for the formation of ^•^CH_2_Cl radicals. Importantly, the system displayed broad substrate scope, including acrylates, acrylamides, acrylonitrile, and dehydroamino acid derivatives, with excellent chemoselectivity and functional group tolerance. Cyclopropanation of substrates bearing protic or amine functionalities, typically problematic in conventional photoredox methods, was achieved efficiently. Beyond CH_2_Cl_2_, the protocol was extended to isotopically labeled CD_2_Cl_2_, enabling direct access to deuterated cyclopropanes of medicinal relevance, and to 1,1-dichloroethane, affording methyl-substituted cyclopropyl adducts.

**Scheme 16:**
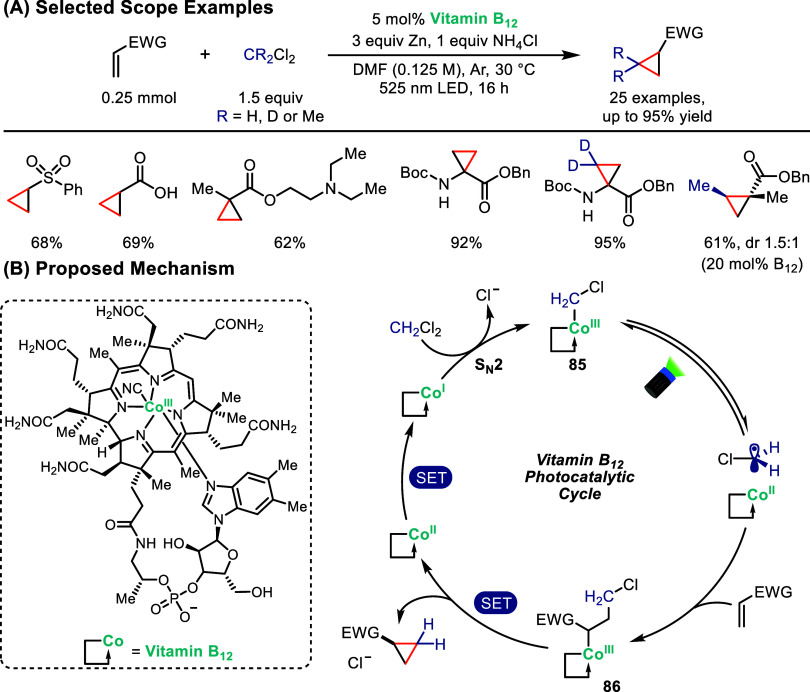
Cobalt-Photocatalyzed Cyclopropanation of Electron-Deficient Alkenes

Over the years, Gryko and co-workers have leveraged the unique nucleophilic and photochemical properties of vitamin B_12_ and analogous complexes for carbon radical generation from a range of precursors, including alkyl halides,^85–87^ strained electrophiles,^88,89^ and diazo compounds.^90^ In a recent notable example, they explored the utility of this catalytic platform toward regioselective epoxide ring-openings.^91^ Although epoxides are versatile building blocks in the synthesis of nonsymmetrical alcohols, challenges with regioselectivity (branched vs linear) remain, especially for aryl epoxides, which react predominantly at the benzylic position.^92^ In this work, the authors combined vitamin B_12_ with nickel catalysis to develop a dual catalytic system to achieve selective cross-electrophile coupling between epoxides and aryl halides under visible-light irradiation, yielding linear alcohols (Scheme 17). Based on the author’s proposal, the mechanism begins with nucleophilic attack of Co^I^ on the less hindered carbon of the epoxide (**87**), generating alkylcobalamin intermediate **88**. Subsequent Co^III^–C bond photolysis generates primary alkyl radical **89** that enters the nickel cycle along with the aryl halide (**90**) to forge the C–C bond following reductive elimination. This sterically controlled pathway overrides the inherent thermodynamic preference for benzylic radical formation, overriding the preference of aryl epoxides to form branched alcohol products. The methodology was effective with both aryl and aliphatic epoxides and tolerated both electron-rich and electron-poor aryl halides, including challenging heteroaryl substrates. Importantly, the cooperative action of vitamin B_12_ photocatalysis with nickel catalysis established a reactivity inaccessible to nickel alone. Additionally, the authors were able to extend this strategy for radical generation via oxetane ring-opening in a subsequent report.^93^

**Scheme 17:**
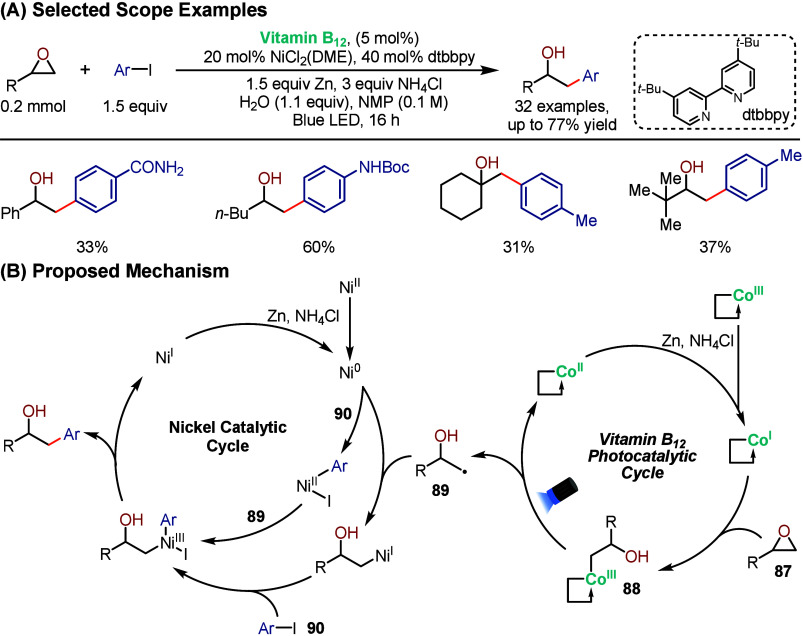
Cobalt and Nickel Dual-Catalyzed Cross-Electrophile Coupling of Epoxides and Aryl Halides

In a follow-up study, Gryko extended this reactivity to aqueous micellar media.^94^ In this work, vitamin B_12_ catalysis facilitates ring-opening of both epoxides and aziridines, followed by radical Giese-type additions to electron-deficient olefins (Scheme 18). Importantly, all products were generated as single regioisomers (linear). This work represents an important advance in reaction sustainability, replacing organic solvents with more environmentally benign aqueous conditions with alcohol cosolvents.

**Scheme 18:**
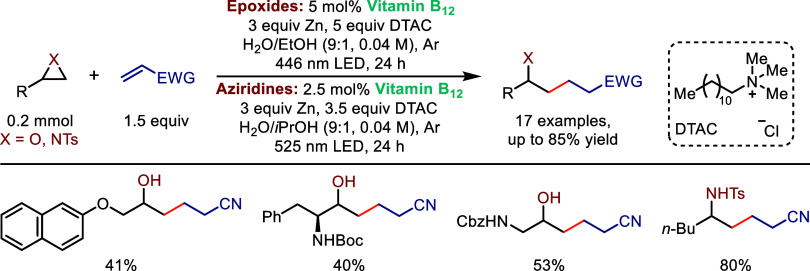
Cobalt-Photocatalyzed Regioselective Ring-Opening of Epoxides and Aziridines in Micellar Solutions

Following the seminal report from Gryko demonstrating the regioselective ring-opening of epoxides using vitamin B_12_, West and co-workers group developed a complementary strategy that converts epoxides directly into Markovnikov alcohols, operating in tandem with thiol-based hydrogen atom transfer (HAT) catalysis (Scheme 19).^95^ The reaction proceeds via initial nucleophilic attack of Co^I^ on the less hindered carbon of the epoxide, furnishing alkylcobalamin intermediate **88** which upon photolysis generates primary alkyl radical **89**. Whereas Gryko’s approach directed this radical into a nickel-mediated cross-electrophile coupling cycle with aryl halides, West’s methodology relies on a TRIP thiol cocatalyst to deliver a hydrogen atom, effectively mimicking hydride nucleophiles and yielding the corresponding alcohol products (**91**) in high regioselectivity. The reaction showed broad functional group tolerance, including electrophilic and reductively labile moieties that would otherwise be susceptible to reduction or cleavage by hydride nucleophiles and the operational simplicity underscores the synthetic potential of this approach. The author proposed that Co^II^ intermediate **92** generated after the radical formation step is key for turning over the thiol catalyst in the presence of a protic solvent. This work represents the first report of demonstration of synergistic vitamin B_12_ photocatalysis and thiol HAT catalysis, and the operational simplicity of the reaction underscores the synthetic potential of this approach.

**Scheme 19:**
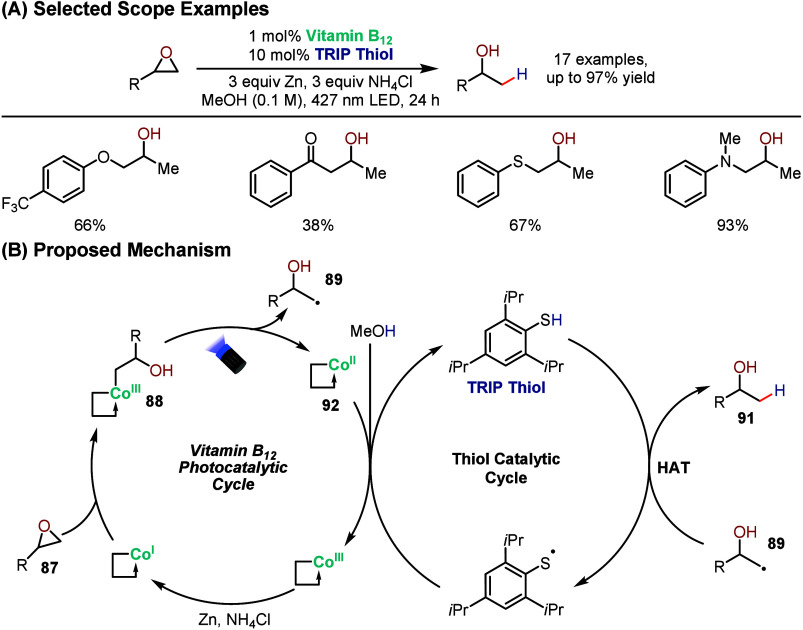
Cobalt and Thiol Dual-Catalyzed Reductive Ring-Opening of Epoxides

### 2.4 Chromium

Chromium is another base metal that has garnered widespread attention in visible-light photocatalysis. Chromium is quite inexpensive, displays low toxicity, and exhibits interesting reactivity, especially Cr^III^ complexes, which benefit from long-lived, highly oxidizing excited states.^96^ Over the years, Ferreira and co-workers have leveraged the unique reactivity of chromium complexes to great effect for a range of reactions, including [4 + 2] and [3 + 2] cycloadditions.^97–100^ In a recent example, they demonstrated that chromium­(III) complexes can photocatalyze the cyclopropanation of alkenes from diazo compounds (Scheme 20).^101^ This scaffold remain difficult to prepare and has been proven useful for preparation of bioactive molecules. Under optimized conditions, they achieved 88% yield of cyclopropyl adduct **94** using only 1 mol % Cr­(Ph_2_phen)_3_]­(BF_4_)_3_ (**93**). The reaction displayed broad functional group compatibility, tolerating TBS protected phenols (**95**), heterocycles (**96**), and sterically demanding substrates (**97**). While trisubstituted alkenes were amenable to the cyclopropanation reaction (**98**), tetrasubstituted alkenes were not tolerated. Additionally, the reaction could also be performed intramolecularly, providing compound **99** in 53% yield. Mechanistically, the authors proposed that the excited **93** oxidizes the alkene **100** to generate radical cation **101**, followed by nucleophilic attack by the diazo compound to generate intermediate **102**. After extrusion of N_2_, intermediate **102** undergoes cyclization to generate radical cation **103**, which undergoes SET to yield the desired cyclopropyl adduct while regenerating the photocatalyst. Cyclic voltammetry experiments were performed to rationalize the feasibility of the electron transfer.

**Scheme 20:**
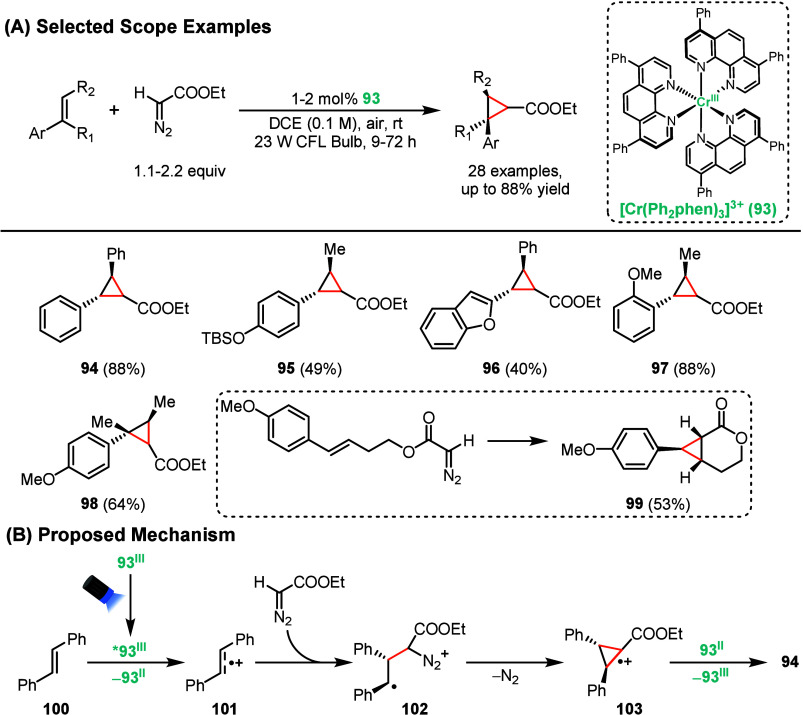
Chromium-Photocatalyzed Cyclopropanation of Alkenes

More recently, Naumann, Heinze, and co-workers published a novel chromium photocatalyst, [Cr­(tpe)_2_]^3+^ (**104**), which could be easily prepared on large scale (>10 g) and could be readily recycled (Scheme 21), representing major improvements in sustainability.^102^ This photocatalyst features spin-flip excited states at an energy of 1.75 eV, and an extremely long excited-state lifetime of 3 ms, making it an attractive option for photocatalysis. In this light, the authors demonstrated the effectiveness of **104** in promoting a diverse range of reactions, including energy-transfer, redox neutral, and net oxidative transformations. For example, they successfully promoted a Newman–Kwart rearrangement, where *O*-aryl thiocarbamate is transformed into *S*-aryl thiocarbamate, generating product **105** in 92% yield at room temperature. This reaction typically requires high temperature (200 to 300 °C), highlighting the significance of this result.^103^ Furthermore, **104** was effective in promoting a radical-cation [4 + 2] cycloaddition, yielding Diels–Alder adduct **106** in 82% yield. Switching the counteranion to nitrate provided a water-soluble variant of **104** while maintaining long excited-state lifetimes, allowing for photoreactions to be conducted under fully aqueous conditions. For example, they demonstrated a decarboxylative fluorination using Selectfluor as the fluoride source using water as the solvent, providing **107** in 80% yield, with the rest of the mass balance corresponding to alcohol **108**. Finally, **104** was found to photocatalyze the radical trifluoromethylation of caffeine starting from sodium trifluoromethanesulfinate in aqueous conditions, providing **109** in 78% yield.

**Scheme 21:**
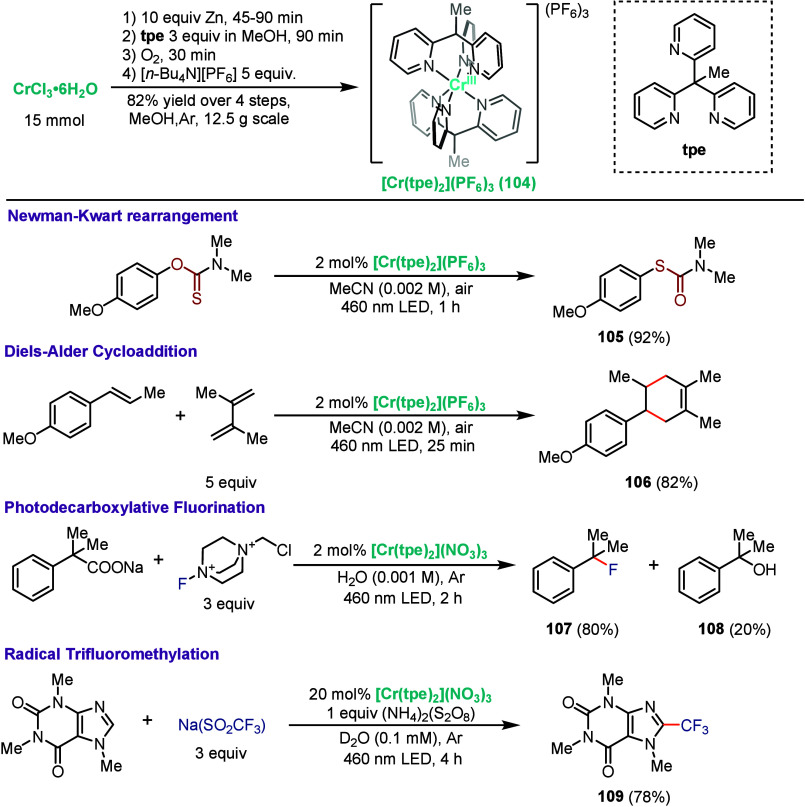
Synthesis and Application of [Cr­(tpe)_2_]^3+^ in Photocatalysis

### 2.5 Manganese

Manganese has recently attracted attention in photocatalysis applications because of its high abundance and low toxicity. The rich redox flexibility of manganese, spanning multiple accessible oxidation states (Mn^0^ to Mn^VII^), provides substantial photocatalytic potential.^104^ These reactive pathways have enabled a variety of transformations, including C–H activation,^105^ oxidative coupling,^106^ and selective functional group modifications.^107^ Despite this, the use of manganese in visible-light-mediated methods remains largely unexplored. Recent progress in applying manganese photocatalysts for light-mediated synthetic methods are highlighted below.

To address the intrinsic limitation of short excited-state lifetimes and limited light absorption in first-row transition metal photocatalysts, Lin and co-workers recently introduced a class of manganese complexes that integrate boron-substituted *N*-heterocyclic carbene ligands (Scheme 20).^108^ These manganese complexes **110** can undergo consecutive excitations across Mn^IV^ and Mn^III^ oxidation states, affording long-lived excited states in the nanosecond-to-microsecond regimes with remarkably strong redox properties. These unique photophysical features enabled efficient photoredox catalysis across both oxidative and reductive manifolds. Most notably, **110** mediated direct, site-selective C–C cross-couplings between arenes and aryl bromides at catalyst loadings as low as 0.5 mol %, with broad functional group compatibility encompassing electron-rich and electron-deficient substrates. Mechanistic studies revealed that the availability of two sequential excited states provides complementary reactivity: the excited *Mn^III^ state serves as a potent reductant capable of aryl halide activation to yield **111**, while the longer-lived excited *Mn^IV^ state acts as a strong oxidant, enabling direct arene oxidation to give **112**. Radical combination between **111** and **112** gives a carbocation intermediate, **113**, which furnishes the coupled product upon deprotonative aromatization (***path a***). Although (***path b***) is energetically less favorable, the abundance of the arene substrate can lead to direct radical addition followed by SET to *Mn^IV^ to also generate **113**. Beyond biaryl construction, the manganese complexes also proved competent in reductive couplings to form C–P, C–B, C–S, and C–Se bonds, as well as oxidative C–N couplings, highlighting the broad synthetic potential of these manganese complexes in photocatalysis (Scheme 22C). This work demonstrates that MC excited states of high-valent manganese can be deliberately engineered to enable powerful and general photoredox chemistry through sequential excitation of multiple oxidation states, without relying on classical MLCT paradigms or precious metals.

**Scheme 22:**
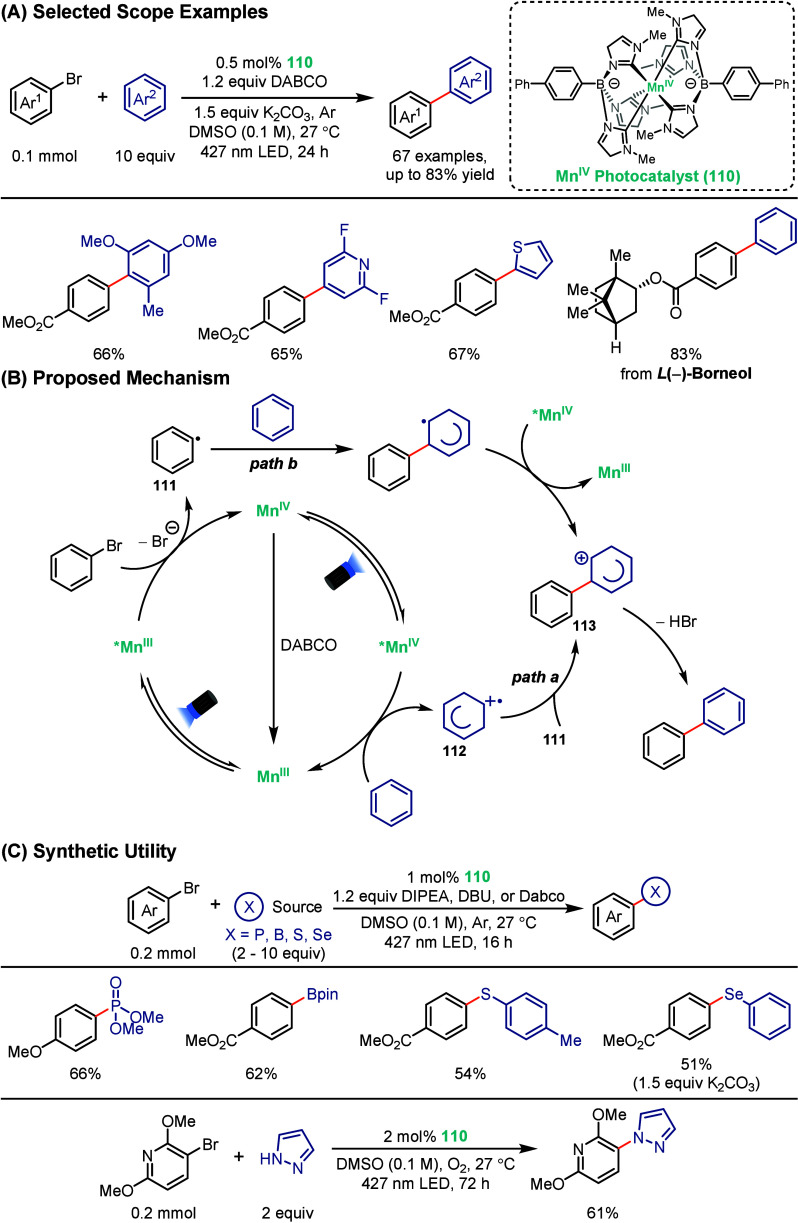
Manganese-Photocatalyzed Cross-Coupling of Arenes

Most recently, Das and co-workers reported an atomically dispersed Mn photocatalyst that represents the first heterogeneous version for vicinal dichlorination of nonactivated alkenes under visible-light irradiation (Scheme 23).^109^ The photocatalyst, Mn_SA_@*f*-C_3_N_4_ (**114**), was prepared by anchoring Mn single atoms onto aryl-amino-functionalized graphitic carbon nitride, offering operational simplicity, recyclability, and minimal manganese loading while maintaining high catalytic activity. Using *N*-chlorosuccinimide (NCS) as a mild chlorinating agent, this system efficiently converted a broad array of alkenes, including terminal, internal, and cyclic substrates, into the corresponding *vic*-dichlorides. The methodology displayed tolerance to diverse functional groups and enabled late stage dichlorination of pharmaceuticals and natural products, underscoring its synthetic relevance. Radical trapping and radical clock experiments provided support the involvement of ^•^Cl intermediates, while multiscale modeling and spectroscopic analyses confirmed that the Mn sites stabilize transient radicals and facilitate selective C–Cl bond formation. The catalyst could be recovered and reused across multiple cycles with only minimal loss in photocatalytic activity, and gram-scale transformations further demonstrated the potential of this platform for sustainable photocatalysis.

**Scheme 23:**
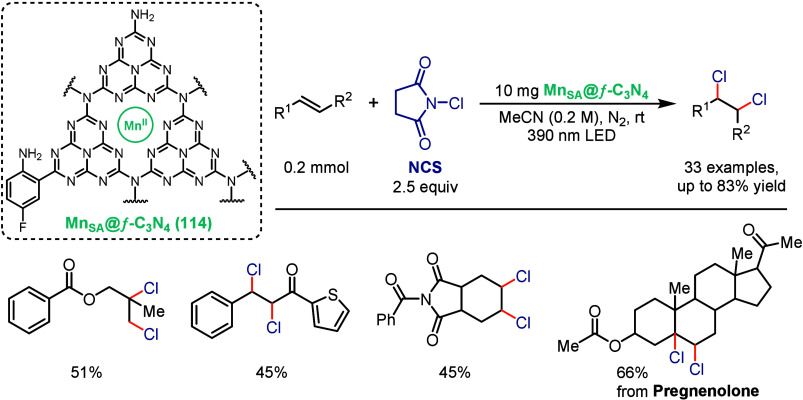
Manganese-Photocatalyzed Vicinal Dichlorination of Alkenes

### 2.6 Vanadium

Vanadium is one of the most abundant base metals, making it an attractive option for developing catalytic platforms. While vanadium–oxo complexes have been widely employed in catalysis for oxidative reactions,^110^ such as the epoxidation of allylic alcohols,^111,112^ their application in photocatalysis remains limited. The following examples serve to highlight the recent progress of vanadium–oxo complexes in oxidative photoredox transformations, leveraging LMCT states to promote unique C–C bond cleavage reactions. The reactions in this section mainly proceed through inner-sphere reactivity, where substrate coordination to vanadium enables LMCT under visible-light irradiation.

In 2019, Wang and co-workers reported a visible-light-driven strategy for selective C–C bond cleavage in lignin model compounds and real lignin extracts using vanadium-based photocatalysts (Scheme 24).^113^ This work was motivated by the challenge of selectively cleaving lignin C–C bonds under mild, additive-free conditions, focusing on the difficult Cα–Cβ bond scission in β-1 and β-O-4 linkages. Vanadium complexes, particularly VO­(O*i*Pr)_3_ and VO­(acac)_2_, showed high catalytic activity under 455 nm irradiation in acetonitrile or acetone/methanol solvents. VO­(O*i*Pr)_3_ achieved near-complete conversion of β-1 model substrates at room temperature with high selectivity for aromatic aldehydes, while VO­(acac)_2_, especially in combination with *N*-hydroxyphthalimide (NHPI), exhibited superior reactivity for β-O-4 model compounds and facilitated higher yields in lignin depolymerization experiments. Mechanistic studies indicate that the lignin substrates first coordinate to the vanadyl center, after which photoexcitation generates an LMCT excited state. This LMCT event enables single-electron transfer (SET) from the bound lignin to vanadium, concomitantly forming benzyl radical intermediates. These radicals then undergo Cα–Cβ bond scission, producing aromatic aldehydes such as vanillin, syringaldehyde, and *p*-hydroxybenzaldehyde. Applied to organosolv lignins under ambient conditions, the vanadium photocatalysts successfully generated aromatic aldehydes, albeit in modest yields (∼0.5–1.4 wt %).

**Scheme 24:**
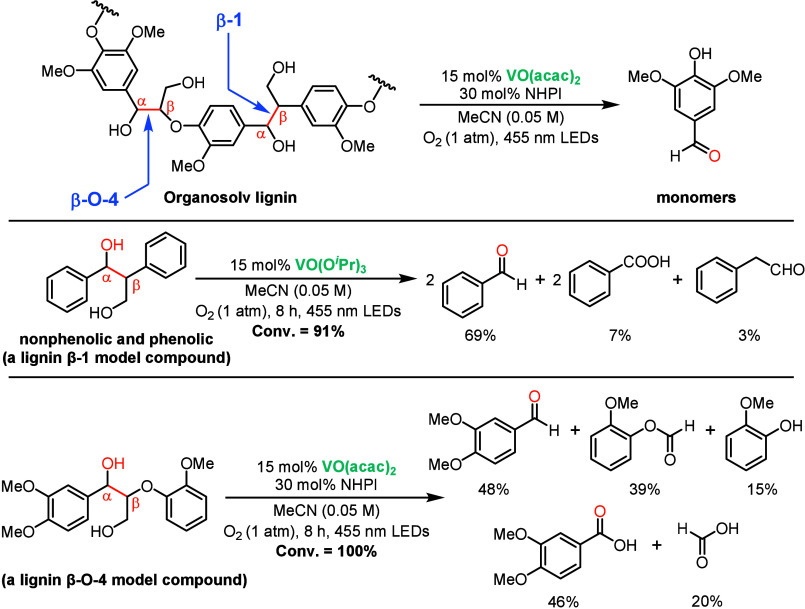
Vanadium-Photocatalyzed Oxidative Lignan C–C Bond Cleavage

In 2023, Soo and co-workers reported the use of a vanadium–oxo photocatalyst containing a redox-noninnocent hydrazone–imidate ligand (**115**) for the deconstructive C–C bond cleavage and functionalization of acyclic and cyclic aliphatic alcohols (Scheme 25).^114^ This work was motivated by the challenge of selectively cleaving inert C­(sp^3^)–C­(sp^3^) bonds in unactivated alcohols under mild conditions, prompting the development of an earth-abundant, visible-light-driven vanadium photocatalytic strategy for deconstructive C–C bond cleavage and late-stage functionalization. Mechanistically, **115** coordinates with the alcohol substrate to generate the key photoactive catalytic intermediate, which undergoes an inner-sphere LMCT induced homolytic C–C bond cleavage upon visible-light irradiation.^115^ Leveraging this reactivity, the authors demonstrated a broad range of functionalization reactions. For example, in the presence of electrophilic radical trapping agents such as DBAD or electron-deficient alkenes, alcohols could be converted to the C–N and C–C coupled products **116** and **117**, respectively, in good yields. For the C–C coupling reactions, 9,10-diphenylanthracene was added as a cophotocatalyst to facilitate catalytic turnover of **115**. In the presence of diethyl bromomalonate (**118**) and hypervalent iodine oxidant **119**, alkyl bromide **120** could be generated in good yield. Reduced product **121** could also be obtained by merging the vanadium photocatalytic cycle with a HAT catalytic cycle using a thiol cocatalyst and Ph_3_SiH as a hydrogen atom source. These deconstructive C–C bond cleavage and functionalization reactions could also be applied to alcohol-containing biomolecules and oligopeptides, highlighting the potential for this approach for late-stage modification of complex molecules.

**Scheme 25:**
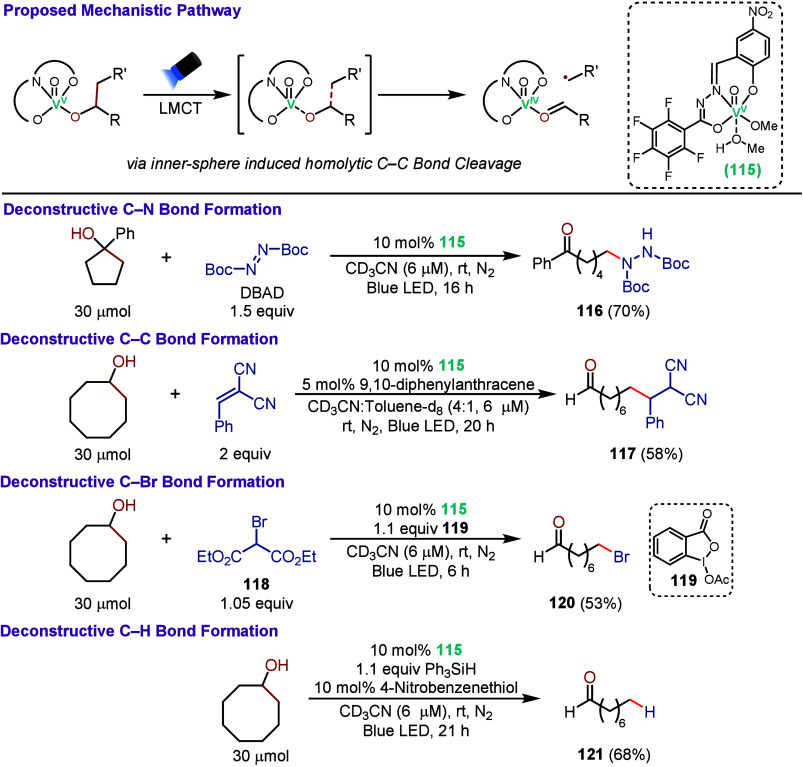
Vanadium-Photocatalyzed C–C Bond Cleavage and Functionalization of Aliphatic Alcohols

## 3 Base Metal Photocatalyst Comparison

In an effort to aid the reader in selecting an appropriate base metal photocatalyst for a given application, we have summarized key properties and reactivity trends for each metal in Table 1. Copper remains one of the most well-studied and versatile base metals for photocatalysis, capable of both outer sphere electron-transfer reactions from MLCT excited states and inner-sphere LMCT reactivity.^52,55,57–60,62^ Additionally, copper complexes have also been shown to mediate XAT reactions.^57,62^ Iron photocatalysis generally proceeds via inner-sphere LMCT and is typically limited to carboxylic acids or alcohols as substrates.^66,71–74^ Cobalt photocatalysts generally exhibit LMCT excited states which can react either via inner or outer sphere mechanisms.^78,79,81,84,91,94,95^ The majority of cobalt photocatalyzed transformations generally proceed under net reductive conditions and represent an excellent choice for generating radicals from halides, pseudohalides and strained electrophiles. Chromium photocatalysts remain one of the best choices for challenging oxidative reactions and have recently been shown to be effective for energy transfer processes.^101,102^ Manganese photocatalysts generally react through an outer-sphere and show great potential for oxidative quenching photocatalytic transformations.^108,109^ Finally, recent examples have demonstrated that vanadium photocatalysts show great promise for mediating inner-sphere induced C–C bond homolysis starting from alcohols and lignan-based substrates, providing a unique mechanistic paradigm that is not readily accessible using other photocatalysts.^113,114^

**Table 1:** Comparison of Properties and Reactivities of Base Metal Photocatalysts Described in This Tutorial Review^a^

base metal	excited state	electron-transfer mechanism	common oxidation states	reaction types	irradiation wavelengths used
copper	MLCT, LMCT	inner or outer sphere	Cu^I^/Cu^II^	redox, XAT	390–530 nm
iron	LMCT	inner sphere	Fe^II^/Fe^III^	redox	390–405 nm
cobalt	LMCT	inner or outer sphere	Co^I^/Co^II^/Co^III^	redox	427–525 nm
chromium	MC SF	outer sphere	Cr^II^/Cr^III^	redox, ET	390 nm
manganese	MC, LMCT	outer sphere	Mn^II^/Mn^III^/Mn^IV^	redox, XAT	390–427 nm
vanadium	LMCT	inner sphere	V^IV^/V^V^	redox	450 nm

a Abbreviations: MLCT: metal-to-ligand charge transfer; LMCT: ligand-to-metal charge transfer; MC: metal-centered; SF: spin-flipped; XAT: halogen atom transfer; ET: energy transfer.

## 4 Summary and Outlook

In recent years, significant progress has been made in leveraging earth-abundant base metal in photoredox catalysis. Despite growing interest, the integration of base metal photocatalysts has remained limited, primarily because of their short excited-state lifetimes and therefore low photocatalytic efficiencies. However, several key strategies to address these shortcomings have emerged, such exploiting the Marcus inverted region,^78^ relying on spin-flip excited states,^102^ or leveraging inner-sphere LMCT for substrate activation.^72,84,114^ Taken together, the examples highlighted in this tutorial review provide sustainable alternatives for promoting a wide range of photoredox transformations. Given the rapid growth of this field, we anticipate further breakthroughs that will increase the library of photoactive base metal complexes, as exemplified by the recent progress in identifying photoactive nickel complexes.^116–118^

While the implementation of base metals represents an important step toward more sustainable photocatalysis, the ligands themselves cannot be ignored, as several base metal photocatalysts still rely on elaborate ligands that require time-consuming synthesis, which inevitably increases their environmental impact. To decrease the reliance of the field on precious metal ruthenium and iridium photocatalysts, which generally rely on simple bipyridine ligands, the continued development of new ligand scaffolds that can be prepared efficiently with inexpensive materials will continue to lower the barrier for the use of base metals in photoredox transformations moving forward.
